# Digital peer support interventions for people with mental health conditions in outpatient settings: a systematic review and meta-analysis

**DOI:** 10.1136/bmjment-2025-302275

**Published:** 2026-02-25

**Authors:** Sarah Croke, Natasha Tyler, Chloe-Nicole Low, Evgenia Gkintoni, Ioannis Angelakis, Ozlem Eylem-Van Bergeijk, Alexander Hodkinson, Brian Mcmillan, Maria Panagioti

**Affiliations:** 1 NIHR School for Primary Care Research, Division of Population Health, Health Services Research and Primary Care, The University of Manchester, Manchester, UK; 2 University of Nottingham School of Medicine, Nottingham, UK; 3 University General Hospital of Patras and Department of Medicine, University of Patras, Patras, Greece; 4 Primary Care and Mental Health, University of Liverpool, Liverpool, UK

**Keywords:** Mental Health, Mental Health Services, Psychiatry, Psychology

## Abstract

Background

Mental health conditions are a major global challenge with rising demand for accessible, effective, scalable treatments. Digital peer support interventions are a promising way to extend support beyond clinical settings, but their effectiveness requires comprehensive evaluation.

Objective

To evaluate the effectiveness of digital peer support interventions in improving clinical symptoms, functioning, and treatment engagement among individuals with mental health conditions in outpatient settings.

**Study selection and analysis:**

We conducted a systematic review and random-effects meta-analysis of controlled interventional studies. Five databases (MEDLINE, CENTRAL, Embase, PsycINFO) were searched up to January 2025. Studies evaluated digital peer support via online platforms, mobile apps or digital communities for people aged ≥16 years with mental health conditions. Outcomes included clinical symptoms (depression, anxiety), functioning (quality of life, social functioning) and treatment engagement. Risk of bias was assessed using Cochrane Risk of Bias 2.0 for randomised controlled trials and ROBINS-I for non-randomised studies. Certainty of evidence was assessed using GRADE (Grading of Recommendations Assessment, Development and Evaluation).

**Findings:**

29 studies including 5825 participants were included. Digital peer support was associated with small-to-moderate improvements in symptoms of depression (standardised mean difference (SMD) −0.28; 95% CI −0.42 to −0.14) and anxiety (SMD −0.47; 95% CI −0.68 to −0.27). Functional outcomes improved modestly: social functioning (SMD 0.18; 95% CI 0.07 to 0.29), quality of life (SMD 0.14; 95% CI 0.02 to 0.26), patient activation (SMD 0.39; 95% CI 0.23 to 0.55) and personal recovery (SMD 0.23; 95% CI 0.11 to 0.35). No significant effects were observed for treatment engagement or satisfaction. Preliminary evidence suggested sustained benefits for depression, anxiety and social functioning.

**Conclusions and clinical implications:**

Digital peer support offers modest improvements in symptoms and functioning for individuals with mental health conditions and may be considered as an adjunct to usual care to enhance engagement and provide accessible support between clinical contacts. Key priorities include establishing optimal intervention models, clarifying longer-term benefits, and ensuring these approaches can be delivered safely and sustainably within routine outpatient services.

**PROSPERO registration number:**

CRD42023445194.

**WHAT IS ALREADY KNOWN ON THIS TOPIC**

- Peer support is a valuable component of mental health care, enhancing personal recovery and engagement.
- Digital technologies are increasingly used to deliver mental health interventions, but the effectiveness of digital peer support in outpatient settings has not been comprehensively evaluated.

**WHAT THIS STUDY ADDS**

- Digital peer support interventions lead to small-to-moderate improvements in depression, anxiety, social functioning and patient activation.
- Interventions delivered by peers alone appear to achieve similar outcomes to those delivered jointly with professionals, though evidence on safety and long-term effects remains limited.

**HOW THIS STUDY MIGHT AFFECT RESEARCH, PRACTICE OR POLICY**

- Supports the integration of digital peer support into outpatient mental health services.
- Emphasises the importance of training, supervision and monitoring to ensure safe and effective delivery while improving access and reducing system burden.

## Introduction

 Mental health conditions affect millions of people worldwide, with around 13% of the population affected each year, and generate economic, human and health costs running into the billions through sickness absence, unemployment, reduced quality of life, premature mortality and healthcare and informal care costs.^1 2^ In response, policymakers increasingly emphasise a shift from inpatient to outpatient care, with a focus on prevention and early intervention to improve access and reduce pressure on acute services.^3 4^

Digital technologies are now widely used in mental healthcare due to their accessibility, affordability and convenience.^5^ These technologies enable remote delivery of peer support through platforms such as social media and mobile applications.^6^ Peer support plays a key role in personal recovery,^7^ involving individuals with shared lived experience and grounded in mutual respect and shared responsibility.^8^ Evidence shows that peer support can enhance traditional treatments and support recovery by improving engagement, health outcomes and self-management.^9 10^ Digital peer support can extend these benefits beyond clinical settings, increasing participation and helping reduce distress and isolation, particularly during periods of uncertainty or transition.^6 11^

Digital peer support interventions include both live and automated formats. While these approaches increase reach, digital delivery may alter relational aspects central to peer support, such as mutuality, shared experience and modelling of hope, particularly where communication is asynchronous or visual and cultural cues are limited.^12^ These differences may influence both effectiveness and implementation and should be considered when evaluating digital peer-supported interventions.

Engagement with digital mental health interventions varies according to symptom severity and individual characteristics, underscoring the need for tailored approaches.^6 11^ Integrating peer support within digital interventions may improve engagement and adherence by fostering trust, strengthening therapeutic alliance and addressing motivational barriers.^13^ In this way, digital peer support may help bridge treatment gaps and support sustained participation in outpatient care.

Despite growing interest, evidence on the effectiveness of digital peer support remains fragmented. Existing reviews have either focused on digital mental health interventions more broadly without isolating peer support^14^,^16^ or have examined digital peer support within specific populations, such as people with severe mental illness or young people, often without meta-analysis.^6 17 18^ There is therefore a need for a focused synthesis of evidence on digital peer support interventions in outpatient mental health settings.

This systematic review and meta-analysis aims to evaluate the effectiveness of digital peer support interventions for people with mental health conditions in outpatient care, examining effects on clinical symptoms, functioning and treatment engagement.

## Methods

This systematic review and meta-analysis was conducted in accordance with Preferred Reporting Items for Systematic Reviews and Meta-Analyses (PRISMA) guidance.^19^ The protocol was registered with PROSPERO (CRD42023445194), the PRISMA checklist was completed and the full protocol is provided in online e-supplemental 1.

### Eligibility criteria

Studies were eligible if they met the following criteria:

- *Population*: Individuals aged 16 years or older with a confirmed or probable mental health condition, identified through clinical interview or validated self-report measures, including schizophrenia, bipolar disorder, depressive and anxiety disorders, obsessive–compulsive disorder, trauma-related disorders, eating disorders and personality disorders.
- *Intervention*: Digital interventions in which peer support was the sole or a major component, including peer-delivered and peer–professional delivered interventions.
- *Comparator*: Any comparator, including usual care, waiting list or no treatment.
- *Outcomes*: Clinical symptoms (eg, depression, anxiety), functioning (overall and social functioning, self-efficacy, personal recovery), treatment engagement (patient activation, service satisfaction, adherence) and adverse events or harms.
- *Design*: Controlled interventional studies, including randomised controlled trials (RCTs), quasi-randomised trials, controlled before-and-after studies and interrupted time series studies.
- *Setting*: Outpatient settings, including primary care and community or social care.

Studies were excluded if they were grey literature, conference abstracts or non-English publications. Studies were also excluded if peer support was a minimal or indirectly implied component of a multicomponent intervention, or if the primary focus was lifestyle change (eg, weight management or physical activity).

### Search strategy and study selection

Five databases (MEDLINE, CENTRAL, Embase and PsycINFO) were searched with no restrictions on follow-up length, language or publication date up to January 2025. Full search strategies are provided in online e-supplemental 2. Reference lists of included studies and relevant reviews were screened, and experts were consulted to identify additional studies.

Search results were uploaded to Covidence (2022). Title screening was piloted on a random sample of 200 records by pairs of reviewers to ensure consistent application of eligibility criteria. A single reviewer then screened titles, excluding clearly irrelevant records. Abstract and full-text screening were conducted independently by two reviewers, with disagreements resolved through discussion or third-party adjudication.

### Data extraction

A standardised data extraction form was developed in Microsoft Excel to collect information on study design, setting, recruitment, baseline characteristics, intervention type (peer-delivered or peer–professional delivered) and outcomes, including clinical symptoms, quality of life, functioning, service satisfaction and treatment engagement. Data on co-design, safety and costs were extracted post hoc. Discrepancies were resolved through discussion or third-party adjudication.

### Risk of bias and certainty of evidence

Risk of bias was assessed using the Cochrane Risk of Bias 2.0 tool for randomised trials and the ROBINS-I tool for non-randomised studies. Blinding items were excluded, as blinding is rarely feasible in talking-based mental health interventions. Disagreements were resolved by consensus or third-party adjudication. Overall certainty of evidence was assessed using the Grading of Recommendations Assessment, Development and Evaluation (GRADE) approach.

### Data analysis

Random-effects meta-analyses were conducted to estimate intervention effects at postintervention. Sensitivity analyses included restricting analyses to studies at low risk of bias and to studies reporting follow-up outcomes beyond 6 months. One prespecified subgroup analysis compared peer-delivered interventions with peer–professional delivered interventions. All analyses were conducted in Stata V.16 using the *metaan* command.^20^ Summary estimates were reported with 95% CIs using the Hartung-Knapp method. Heterogeneity was assessed using forest plots, prediction intervals and I² and τ² statistics.^21^ Where 10 or more studies were available, funnel plots and Egger tests were used to assess small-study effects.^22 23^ Cluster-randomised trials were adjusted using an inflation factor assuming an intraclass correlation coefficient of 0.02. Studies with multiple peer support arms were treated as independent comparisons, with control groups split accordingly.

## Results

**Figure 1:**
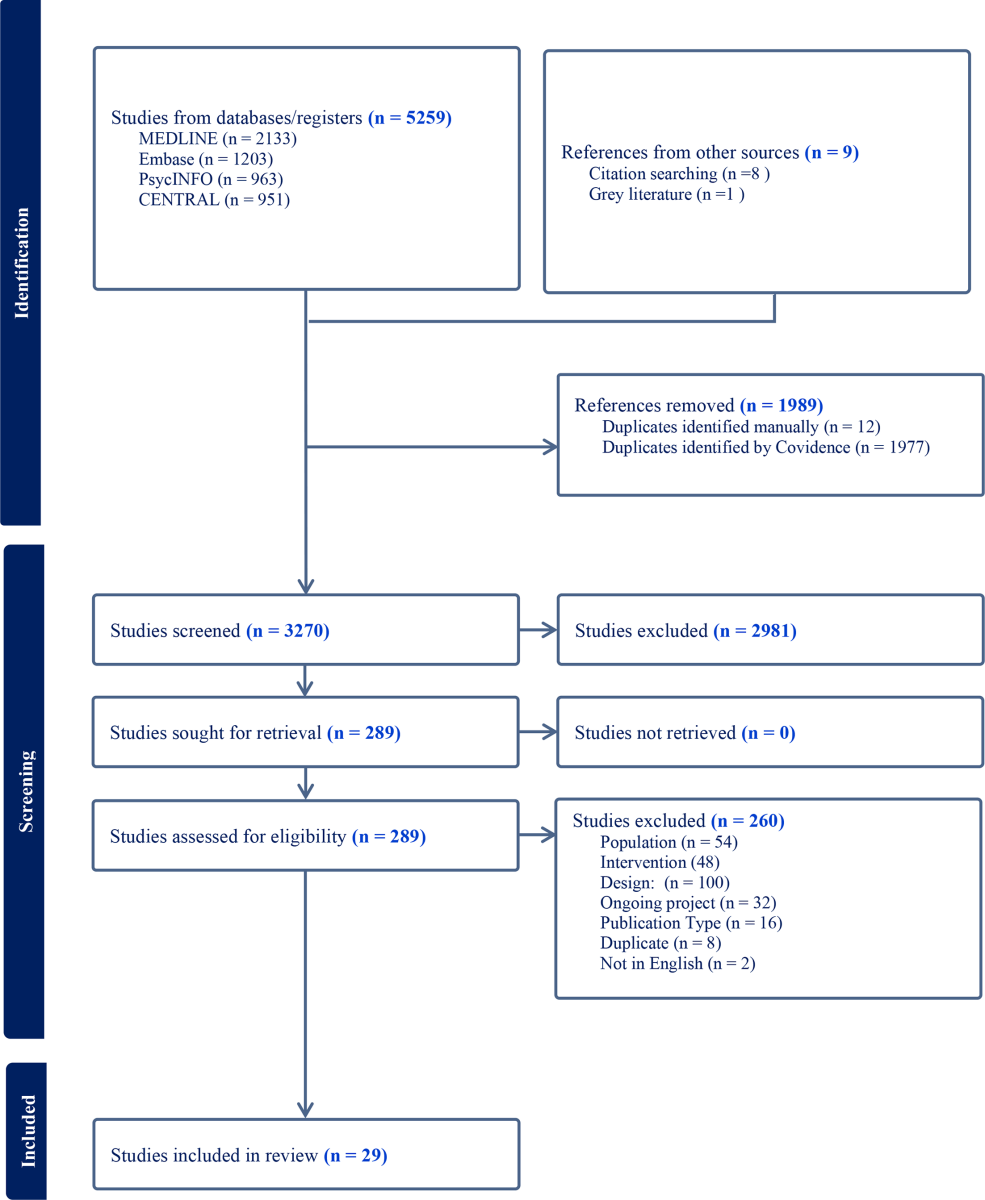
PRISMA flow diagram. PRISMA, Preferred Reporting Items for Systematic Reviews and Meta-Analyses.

The searches identified 5259 records which, following deduplication, title and abstract screening and full-text screening, resulted in 29 studies included in this review. The full reference list of included studies is provided in online e-supplemental 3 to comply with the journal guideline limiting the number of references to 50. Of these, 26 were included in the meta-analysis and 3 were synthesised narratively. Figure 1 presents the PRISMA flow diagram.

### Descriptive characteristics of the included studies

The 29 included studies were conducted across 9 countries, including two international collaborations. Among single-country studies, the majority were conducted in the USA (14 studies; 52%), followed by Australia (4 studies; 15%) and Canada (3 studies; 11%). Austria, Germany, Japan, the Netherlands, Singapore and the UK each contributed one single-country study (4% each). 24 studies (83%) were based in community services, 3 (10%) in primary care settings and 2 (7%) in hospital-to-community transition settings. 23 of the included studies (79%) were full-scale RCTs, 4 studies (14%) were pilot or feasibility RCTs and the remaining 2 studies (7%) were non-RCT controlled interventions.

The total number of participants across the included studies was 5825, with sample sizes ranging from 20 to 2480 (median n = 138). Among the 26 studies that reported mean age, the average was 35 years, with a median of 39 years and a range of 18–70 years. 20 studies included predominantly female participants (over 50% of the sample), 8 included predominantly male participants and 1 did not report the gender composition. Of the 29 studies, 9 (31%) included participants with serious mental illness (eg, schizophrenia, schizoaffective disorder or bipolar disorder), 17 (59%) focused on common mental health conditions (eg, depression, anxiety disorders, post traumatic stress disorder) and 3 (10%) involved people with mixed or unspecified conditions.

Almost half of the interventions (14; 48%) involved peer support delivered without input from health professionals—for example, through trained peer counsellors. The remaining interventions (15; 52%) combined peer support with professional involvement. 22 studies (76%) delivered peer support exclusively through online platforms, while 7 (24%) used blended approaches combining online components with face-to-face and/or telephone-based peer support. Only six studies involved end users or peer workers in the design, development or delivery of interventions. Control conditions varied across studies: 9 (31%) used treatment as usual, 8 (28%) provided enhanced usual care (eg, booklets, websites), 10 (34%) employed waitlist or delayed-intervention controls and 2 (7%) offered no treatment.

Only one conducted a formal economic evaluation, showing significantly lower costs and improved social functioning for the intervention compared with treatment as usual.^24^ Safety data were reported in five studies, with adverse events ranging from minor to serious, including suicide attempts or self-injury, but no intervention-related deaths were recorded.

More detailed information on the characteristics of the studies and interventions is available in the online supplemental eTables 1 and 2.

### Risk of bias results

Of the 29 studies, 13 (45%) were at high risk of bias and 16 (55%) at low-to-moderate risk. Excluding blinding, which was a universal issue (93%), common issues included high attrition or incomplete data (31%), selective or unclear reporting (24%) and inadequate randomisation procedures (41%). Detailed scores are provided in online supplemental eTable 3.

### Meta-analysis results

Data from 26 of the 29 eligible studies were pooled in a meta-analysis to evaluate the effectiveness of peer support interventions across three domains: clinical symptoms, aspects of functioning and treatment engagement.

### Clinical symptoms

**Figure 2:**
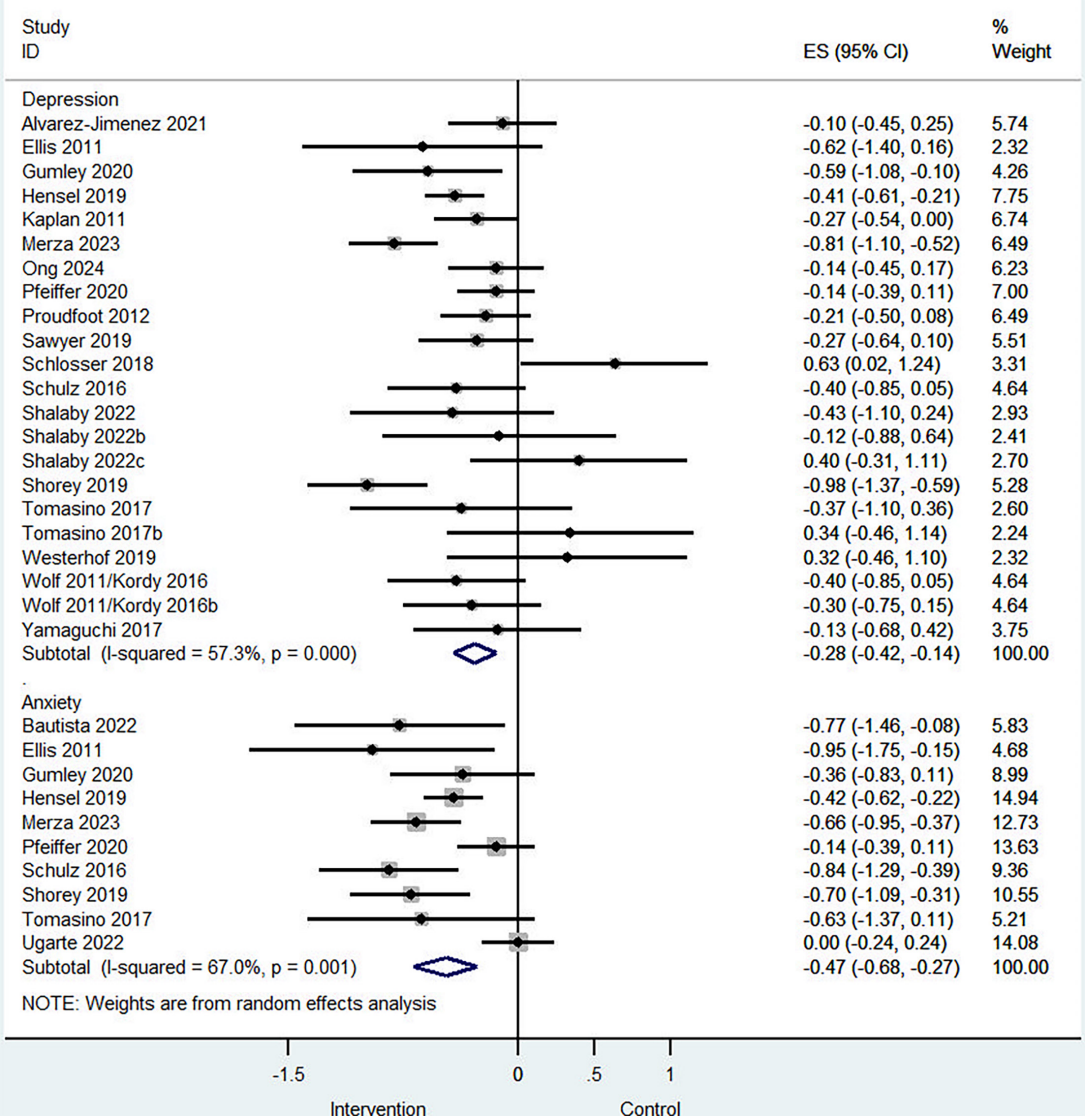
Forest plot of the effects of peer support interventions on clinical symptoms.

22 studies assessed peer support interventions for depression symptoms. Compared with controls (typically treatment as usual), peer support was associated with a small-to-moderate reduction in depression symptoms (standardised mean difference (SMD) –0.28; 95% CI –0.42 to –0.14; I² = 57%; p<0.001; figure 2), with moderate heterogeneity observed. Egger’s test indicated no evidence of small-study effects (intercept, 1.06; SE, 0.81; p=0.21; online supplemental eFigure 1). Excluding studies at high risk of bias yielded similar results (SMD –0.29; 95% CI, –0.44 to –0.14; I² = 57%; p<0.01; online supplemental eFigure 2). Five studies reporting long-term depression outcomes (≥12 months) showed a significant effect (SMD –0.21; 95% CI –0.38 to –0.04; online supplemental eFigure 3), suggesting that reductions were sustained. Peer-delivered interventions were associated with greater reductions in depression symptoms (SMD –0.36; 95% CI –0.63 to –0.10; I² = 73%; p<0.001) than interventions delivered by both peers and professionals (SMD –0.24; 95% CI –0.38 to –0.09; I² = 34%; p=0.11), though the between-group difference was not statistically significant (online supplemental eFigure 4).

10 studies evaluated peer support interventions for anxiety. Compared with controls, interventions were associated with a moderate reduction in anxiety symptoms (SMD –0.47; 95% CI –0.68 to –0.27; I² = 67%; p<0.01; figure 2), with high heterogeneity observed. Egger’s test did not indicate small-study effects (intercept, –2.20; SE, 1.16; p=0.09; online supplemental eFigure 5). Excluding studies at high risk of bias yielded similar results (SMD –0.45; 95% CI –0.68 to –0.22; I² = 60%; p<0.05; online supplemental eFigure 2). Peer-delivered interventions were associated with greater reductions in anxiety symptoms (SMD –0.51; 95% CI –0.81 to –0.22; I² = 77%; p<0.001) than mixed peer–professional delivered interventions (SMD –0.42; 95% CI –0.60 to –0.25; I² = 34%; p=0.83), though the between-group difference was not statistically significant (online supplemental eFigure 6).

### Aspects of functioning

**Figure 3:**
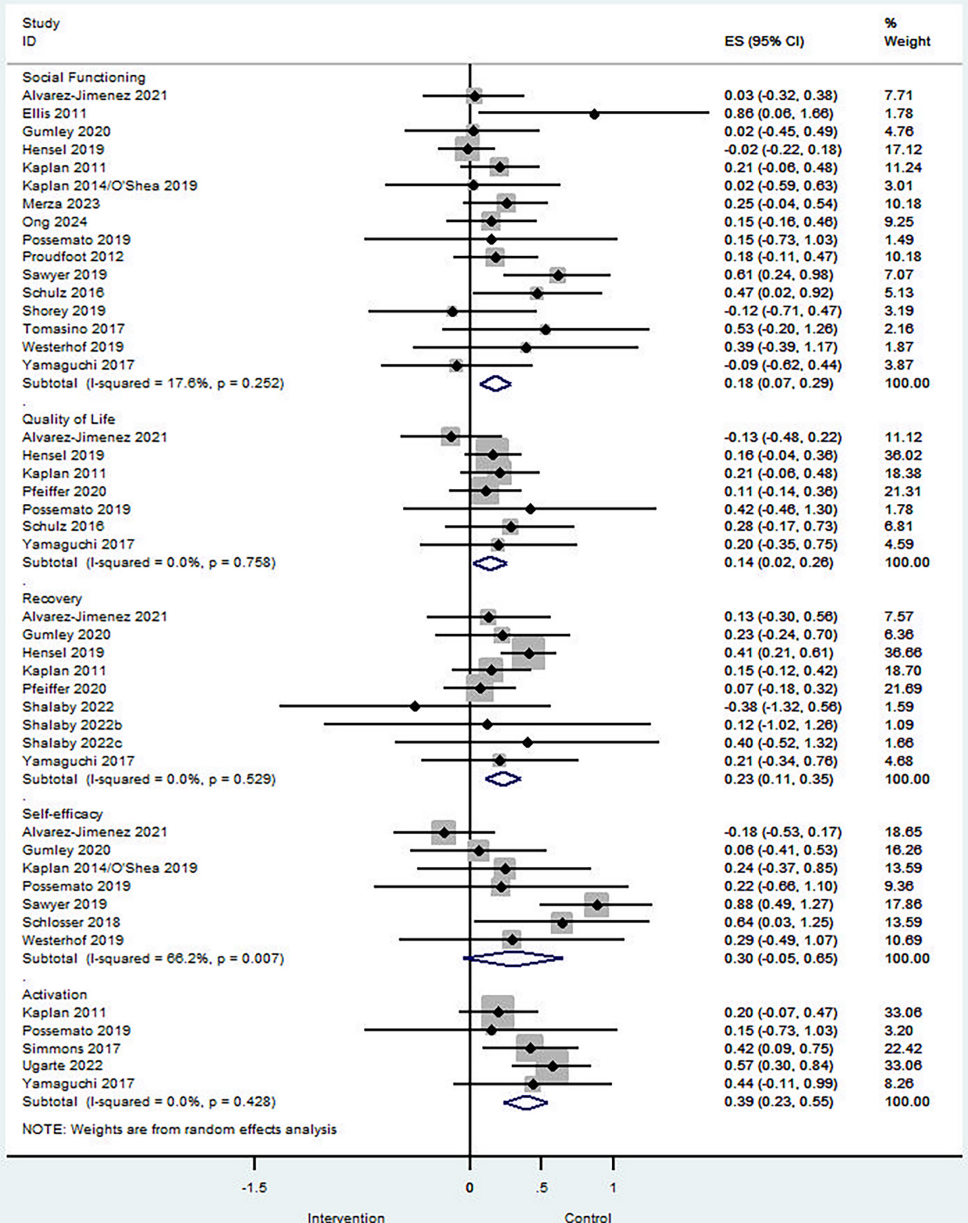
Forest plot of the effects of peer support interventions on aspects of functioning.

16 studies evaluated social functioning and quality of social relationships. Peer support yielded a small but significant improvement (SMD 0.18; 95% CI 0.07 to 0.29; I² = 18%; p=0.25; figure 3) with low, non-significant heterogeneity. Egger’s test did not indicate small-study effects (intercept, 0.91; SE, 0.66; p=0.19; online supplemental eFigure 7). Excluding studies at high risk of bias yielded similar results (SMD 0.15; 95% CI 0.03 to 0.28; I² = 21%; p=0.25; online supplemental eFigure 8). Four studies reporting long-term outcomes (≥12 months) showed sustained improvements (SMD 0.18; 95% CI 0.01 to 0.36; I² = 0%; p=0.738; online supplemental eFigure 9) with no heterogeneity. Peer-delivered interventions were associated with similar improvements in social functioning (SMD 0.20; 95% CI 0.04 to 0.36; I² = 0%; p=0.55) compared with interventions delivered by both peers and professionals (SMD 0.18; 95% CI 0.02 to 0.34; I² = 34%; p=0.11; online supplemental eFigure 10).

Seven studies assessed peer support interventions for quality of life, showing a small but statistically significant positive effect (SMD 0.14; 95% CI 0.02 to 0.26; I² = 0%; p=0.76; figure 3), indicating no heterogeneity across studies.

Nine studies assessed peer support interventions for personal recovery outcomes, demonstrating a small but significant positive effect (SMD 0.23; 95% CI 0.11 to 0.35; I² = 0%; p=0.53; figure 3), with no evidence of heterogeneity.

Five studies investigated in patient activation and empowerment, revealing a moderate, significant positive effect (SMD 0.39; 95% CI 0.23 to 0.55; I² = 0%; p=0.62; figure 3) with negligible heterogeneity.

Seven studies examined peer support interventions for self-efficacy, showing a small, non-significant improvement (SMD 0.30; 95% CI –0.05 to 0.65; I² = 66%; p<0.01; figure 3) with moderate and significant heterogeneity.

### Treatment engagement

Six studies assessed peer support interventions for treatment engagement. Compared with controls, interventions showed a non-significant positive effect (SMD 1.75; 95% CI –1.95 to 5.44; I² = 99%; p<0.001; online supplemental eFigure 11), with high heterogeneity across studies.

Five studies evaluated peer support interventions for satisfaction with services. Compared with controls, interventions did not show a significant effect (SMD 0.13; 95% CI –0.16 to 0.43; I² = 38%; p=0.17; online supplemental eFigure 11), with low and non-significant heterogeneity.

### Narrative synthesis results

Three studies did not provide data amenable to meta-analysis and were instead synthesised narratively. These studies provide broadly consistent insights into the effectiveness of peer support interventions, highlighting short-term benefits but also variability in long-term outcomes and treatment engagement. Kaveladze *et al*^25^ found reductions in depression and anxiety associated with crowdsourced peer support but limited significant effects over time, aligning with the findings of the meta-analysis. Kelly *et al*^26^ reported improved healthcare engagement but no changes in self-management associated with the peer-health navigator intervention. Simon *et al*^27^ observed high initial engagement in the peer support coaching group, followed by substantial drop-offs after 3 weeks, reflecting challenges in sustaining participation as noted in the meta-analysis. Whether this level of exposure is sufficient to produce meaningful effects remains unclear, underscoring the need for research on minimum effective doses.

### GRADE assessment results

The quality of evidence was moderate for key clinical outcomes such as depression and anxiety, as well as for secondary outcomes including functioning, quality of life, personal recovery and patient activation, reflecting some concerns regarding risk of bias and heterogeneity. However, treatment engagement outcomes were supported by low-quality evidence due to imprecision and variability across studies. No strong publication bias was detected. Overall, the findings provide moderate confidence in the effectiveness of digital peer support interventions for improving mental health symptoms and aspects of quality of life and social functioning.

## Discussion

### Summary of key findings

This systematic review indicates that digital peer support interventions can improve mental health outcomes in outpatient settings. Short-term benefits were observed for clinical symptoms, with moderate reductions in anxiety and small-to-moderate reductions in depression, and limited evidence suggests potential longer-term effects. Interventions also improved broader outcomes, including quality of life, social functioning, personal recovery and patient activation. In contrast, effects on treatment engagement were inconsistent and highly variable across studies.

Evidence on sustained outcomes remains limited. Among the few studies reporting long-term effects, all involved peer–professional delivery, largely focused on psychotic disorders and one on major depression, highlighting gaps in evidence across conditions and populations. Peer-delivered interventions produced similar improvements in symptoms and social functioning to those delivered jointly by peers and healthcare professionals. Future research should examine how intervention context, delivery models and mechanisms influence effectiveness and real-world implementation.

### Comparison with previous reviews

Previous reviews of digital mental health interventions report good feasibility and acceptability but mixed or modest effects on clinical outcomes.^14^,^1628^ Broader reviews of digital psychiatry highlight the scalability of technologies such as smartphones, social media, chatbots and virtual reality, while emphasising that effectiveness depends on implementation, engagement and contextual fit within health systems.^5^ A recent large meta-analysis of digital interventions for people with schizophrenia found no significant pooled effects on symptoms, functioning or quality of life, despite high retention.^29^ However, trends favoured interventions with human support, particularly for social cognition and quality of life. In these studies, support was typically clinician-led or facilitator-led rather than peer-delivered, and co-design was inconsistently reported, limiting conclusions about the specific role of peer support.

Reviews focusing specifically on peer support have mainly examined in-person interventions or specific populations, such as people with severe mental illness or young people, and report heterogeneous or limited evidence.^6 17 18 30^ For example, Fortuna *et al*^6^ demonstrated feasibility and acceptability of digital peer support but did not quantify effectiveness, while Ali *et al*^17^ found supportive but limited evidence among young people. Our findings extend this literature by providing quantitative evidence across a broader range of outpatient populations.

This review also highlights suboptimal use and reporting of coproduction in digital peer support interventions.^31^ This reflects wider evidence that, despite strong recommendations, coproduction is inconsistently implemented in digital mental health research. More systematic use of coproduction across design, development and evaluation may improve acceptability, cultural relevance and engagement. Clearer reporting of patient and public involvement is needed to strengthen the evidence base and support safe scale-up.

### Strengths and limitations

Strengths of this review include rigorous methodology, adherence to reporting guidelines, inclusion of studies from diverse settings and use of random-effects meta-analysis with sensitivity analyses. These features strengthen confidence in the observed effects on clinical and social outcomes in outpatient populations.

Several limitations should be noted. The small number of studies in some analyses limited exploration of heterogeneity using methods such as meta-regression. Substantial variation in intervention design, delivery and populations further complicates interpretation and underscores the need to identify effective components. Evidence on long-term outcomes was scarce, with most studies reporting short-term effects only, limiting conclusions about sustainability and subgroup effects. Study quality varied, with some risk of bias, although sensitivity analyses suggested overall robustness. Exclusion of non-English studies and grey literature may have introduced selection bias, and while publication bias was assessed for clinical outcomes, it may still affect other domains.

Few studies reported safety outcomes, which is concerning given known risks in mental health interventions, including deterioration, non-response and dependency.^32 33^ Future studies should prioritise systematic assessment and reporting of safety and harms.

### Implications for policy and practice

Digital peer support has potential to enhance access to outpatient mental healthcare by providing ongoing support beyond face-to-face consultations, which is particularly relevant given increased service demand following the COVID-19 pandemic. These interventions may reduce pressure on health systems while improving symptoms, quality of life, social functioning and personal recovery. However, evidence on cost-effectiveness is limited. Only one study identified in a post hoc search reported a formal economic evaluation, suggesting cost savings compared with treatment as usual. Further economic studies are needed to inform commissioning decisions.^24^

Peer-delivered digital interventions appear as effective as those delivered jointly with healthcare professionals, supporting their potential scalability. However, appropriate supervision and safeguards are essential, particularly given the challenges of peer delivery.^34 35^ Policymakers and clinicians may consider peer-led digital models as a resource-efficient option for individuals facing barriers to in-person care, including during transitions between services. Investment in training peer support specialists in digital skills, engagement, risk monitoring and therapeutic alliance development is critical.^36 37^

Despite their promise, safety reporting was inconsistent, with few studies monitoring adverse events, symptom deterioration or suicidality. Although no clear harms were identified, risks commonly reported in mental health interventions remain relevant.^32 33^ These concerns may be heightened in peer-delivered models, where peers may lack formal clinical training. Future implementation should prioritise routine safety monitoring, transparent reporting and alignment with emerging best-practice guidance.^38^ Improved understanding of safety risks will support protocol development and continuous harm assessment.

Future research should also identify the active ingredients of digital peer support. Core peer mechanisms, such as shared lived experience, emotional validation, empowerment and identity reconstruction, may translate to digital formats, alongside unique features such as anonymity, which may reduce fear of judgement but limit emotional nuance.^12^ Clarifying these mechanisms will support optimisation of intervention design and targeting of processes linked to engagement and recovery.

Finally, cultural and intersectional factors warrant greater attention. Most studies were conducted in Western contexts, yet peer support may function differently in collectivist cultures or among marginalised groups, where norms, power dynamics and structural inequities shape engagement and trust.^39^ Variability in treatment engagement may partly reflect cultural differences in how engagement is understood. Future research should develop culturally responsive and equitable digital peer support models.

## Conclusion

Digital peer support interventions can improve symptoms, social functioning and quality of life in outpatient mental healthcare. Further research should clarify mechanisms of action, identify key components and examine long-term outcomes across diverse populations.

## Supplementary material

10.1136/bmjment-2025-302275 online supplemental file 1

## Data Availability

Data are available upon reasonable request.
