# Supplementary material for: Digital peer support interventions for people with mental health conditions in outpatient settings: a systematic review and meta-analysis
Source: BMJ Ment Health. 2026 Feb 25;29(1):e302275. doi: 10.1136/bmjment-2025-302275 (PMC12958987; doi:10.1136/bmjment-2025-302275)

**eSupplement 1: Digital Peer Support Interventions for People With Mental Illness: Protocol for a Systematic Review and meta-analysis**

**Introduction**

Mental health conditions are highly prevalent, with an estimated 18–36% of the global population experiencing a diagnosable disorder during their lifetime.¹ This burden affects individuals, families, and communities, contributing to economic costs, reduced productivity, and personal distress.²–⁶ For example, the cost of depression in Japan in 2005 was estimated at JPY 2 trillion (USD 18 billion),⁴ and in Europe, the societal costs of brain disorders exceed 800 billion euros annually.⁵ Stigma, limited access to treatment, and underfunded mental health services—particularly in low-income countries—further exacerbate these challenges.³ The WHO has emphasised mental health as a global priority, stating “no health without mental health.”³

Peer support is a core component of mental health recovery, involving support from individuals with lived experience.⁷,⁸ It enhances traditional treatments, increasing engagement, self-management, and empowerment.⁸–¹⁰ Digital technologies now allow peer support to be delivered via apps, online platforms, and social media, either synchronously or asynchronously, extending reach and engagement.¹¹–¹⁴ Digital peer support has shown benefits in symptom management, self-efficacy, hope, and quality of life.¹¹,¹³ These interventions can be co-developed with peers and researchers, promoting evidence-based, user-centred approaches.¹³

Patients often face waiting periods for mental health treatment in primary care and outpatient care, during which distress and isolation can increase. Digital peer support interventions can provide timely support, connecting individuals with peers who share similar experiences, reducing distress, and promoting coping strategies.

**Rationale for this review**

Existing reviews on digital peer support have largely focused on severe mental illness and have not used meta-analysis.¹¹ There is a need for a systematic review assessing the effectiveness of digital peer support interventions across outpatient populations. This review aims to evaluate whether these interventions improve outcomes such as symptom severity, quality of life, patient activation, and treatment adherence.

**Methods**

The systematic review and meta-analysis will be conducted in accordance with published guidelines (Cochrane Handbook¹⁵ and PRISMA¹⁶), and the protocol will be registered with PROSPERO.

**Eligibility criteria**

Studies will be eligible if they meet the following criteria:

- **Population:** People aged ≥16 with a mental illness, including schizophrenia spectrum and other psychotic disorders, bipolar and related disorders, depressive disorders, anxiety disorders, obsessive-compulsive and related disorders, trauma- and stressor-related disorders, eating disorders, and personality disorders.
- **Intervention:** Digital interventions or tools utilising peer support (including peer-delivered, peer-augmented, and peer-to-peer social media interventions).
- **Comparator:** Any comparator (e.g., usual care, waiting list, no treatment) or no comparator (uncontrolled designs).
- **Outcomes:** Improved quality of life, psychiatric symptom severity, patient satisfaction/knowledge/activation, or treatment engagement.
- **Design:** Controlled interventional designs, including RCTs, quasi-randomised trials, controlled before-and-after studies, and interrupted time series studies.
- **Setting:** Outpatient care, primary care or community/social care settings.

**Searching and selection**

We will search Medline, CENTRAL, Embase, PsycINFO, and one additional database (to be specified), without restrictions on follow-up duration, language, or publication date. Reference lists of included studies and relevant systematic reviews will also be screened, and experts consulted for additional studies. An updated search will be conducted prior to submission to capture newly published studies.

Search results will be exported to EndNote for duplicate removal and then uploaded to Covidence¹⁷ for screening. Title screening will be piloted on a random sample (n=200) by pairs of reviewers to ensure consistency. Thereafter, single reviewers will exclude clearly irrelevant studies. Abstract and full-text screening will be performed independently by two reviewers, with disagreements resolved by discussion or adjudication by a third reviewer.

**Data extraction**

A customised data extraction form in Microsoft Excel will capture: study design; setting; recruitment and sampling; aims and inclusion criteria; baseline sample characteristics; intervention type and intensity (peer-delivered, jointly delivered with professionals); and outcomes (quality of life, symptom reduction, patient satisfaction/knowledge/activation, and treatment adherence). Discrepancies will be resolved by a third reviewer.

**Quality appraisal**

Risk of bias will be assessed using the Cochrane Risk of Bias Tool 2.0 for trials¹⁸ and the EPOC checklist for other controlled designs¹⁹. The overall strength of evidence will be evaluated using CINeMA²⁰, considering within-study bias, reporting bias, indirectness, imprecision, heterogeneity, and incoherence.

**Data analysis**

Where suitable, random-effects meta-analysis will be conducted for primary and secondary outcomes. Analyses will be performed separately by study design (RCTs, comparative cohorts, before-after designs). If feasible, subgroup analyses or meta-regressions will explore the impact of intervention type, context, and participant characteristics. Sensitivity analyses will exclude studies at high risk of bias.

All analyses will be conducted in Stata 16 using metaan and metareg commands²¹. Random-effects models will be applied due to their conservative properties in the presence of heterogeneity. Summary estimates will be presented with 95% confidence intervals using the Hartung-Knapp method²².

Heterogeneity will be assessed via forest plots with prediction intervals, and I² and τ² statistics²³. If ≥10 studies are included, funnel plots and the Egger test will assess small-study bias²⁴. Funnel plots will be generated with the metafunnel command, and the Egger test with metabias. For cluster-randomised trials, analyses will be adjusted for clustering using a sample size/variance inflation method, assuming an intraclass correlation of 0.02²⁵.

**References**

1. Kessler RC, Berglund P, Demler O, et al. Lifetime prevalence and age-of-onset distributions of mental disorders in the World Health Organization's World Mental Health Survey Initiative. *World Psychiatry* 2007;6:168–176.
2. Whiteford HA, Degenhardt L, Rehm J, et al. Global burden of disease attributable to mental and substance use disorders: findings from the Global Burden of Disease Study 2010. *Lancet* 2015;382:1575–86.
3. WHO. Depression and other common mental disorders: global health estimates. Geneva: World Health Organization, 2017.
4. Sado M, et al. Cost of depression among adults in Japan. *Clin Drug Investig* 2011;31:717–28.
5. Smith K. European Brain Council launches Atlas of the Brain. *Lancet Neurol* 2011;10:885.
6. Rehm J, et al. The global burden of disease attributable to alcohol and drug use in 195 countries and territories, 1990–2016: a systematic analysis for the Global Burden of Disease Study 2016. *Lancet Psychiatry* 2019;5:987–1012.
7. Solomon P. Peer support/peer provided services: underlying processes, benefits, and critical ingredients. *Psychiatr Rehabil J* 2004;27:392–401.
8. Mead S, MacNeil C. Peer support: what makes it unique. *Int J Psychosoc Rehabil* 2006;10:29–37.
9. Wexler B, Davidson L, Styron T, Strauss J. 40 years of academic public psychiatry. In: Jacobs S, Griffiths EEH, editors. *Severe and persistent mental illness*. Hoboken, NJ: John Wiley & Sons, 2007:1–20.
10. Chinman M, George P, Dougherty RH, et al. Peer support services for individuals with serious mental illnesses: assessing the evidence. *Psychiatr Serv* 2014;65:429–41.
11. Druss BG, Singh M, von Esenwein SA, et al. Peer-led self-management of general medical conditions for patients with serious mental illnesses: a randomized trial. *Psychiatr Serv* 2018;69:529–35.
12. Fortuna KL, Naslund JA, LaCroix JM, et al. Digital peer support mental health interventions for people with a lived experience of a serious mental illness: systematic review. *JMIR Ment Health* 2020;7:e16460.
13. Fortuna KL, Aschbrenner KA, Lohman MC, et al. Smartphone ownership, use, and willingness to use smartphones to provide peer-delivered services: results from a national online survey. *Psychiatr Q* 2018;89:947–56.
14. Fortuna KL, Venegas M, Umucu E, et al. The future of peer support in digital psychiatry: promise, progress, and opportunities. *Curr Treat Options Psych* 2019;6:221–31.
15. Higgins JPT, Thomas J, Chandler J, et al., editors. Cochrane Handbook for Systematic Reviews of Interventions. 2nd edition. Chichester (UK): John Wiley & Sons; 2019.
16. Page MJ, McKenzie JE, Bossuyt PM, et al. The PRISMA 2020 statement: an updated guideline for reporting systematic reviews. BMJ. 2021;372:n71.
17. Covidence systematic review software, Veritas Health Innovation, Melbourne, Australia. Available at: www.covidence.org
18. Sterne JAC, Savović J, Page MJ, et al. RoB 2: a revised tool for assessing risk of bias in randomised trials. BMJ. 2019;366:l4898.
19. Cochrane Effective Practice and Organisation of Care (EPOC) Group. EPOC Resources for Review Authors. Available at: https://epoc.cochrane.org/resources/epoc-resources-review-authors
20. Papakonstantinou T, Nikolakopoulou A, Rücker G, et al. CINeMA: Software for semiautomated assessment of the confidence in the results of network meta-analysis. PLoS One. 2020;15(12):e0242204.
21. Kontopantelis E, Reeves D. Performance of statistical methods for meta-analysis in the presence of heterogeneity and small study effects. Stat Methods Med Res. 2013;22(1):3–22.
22. Hartung J, Knapp G. A refined method for the meta-analysis of controlled clinical trials with binary outcomes. Stat Med. 2001;20:3875–3889.
23. Higgins JPT, Thompson SG, Deeks JJ, Altman DG. Measuring inconsistency in meta-analyses. BMJ. 2003;327:557–560.
24. Sterne JAC, Egger M, Smith GD. Investigating and dealing with publication and other biases in meta-analysis. BMJ. 2001;323:101–105.
25. Campbell MJ, Walters SJ. How to Design, Analyse and Report Cluster Randomised Trials in Medicine and Health Related Research. 2nd edition. Chichester: Wiley, 2014

**e-Supplement 2: Searches**

| Ovid MEDLINE(R) <1946 to June Week 1 2025> | | |
| --- | --- | --- |
|  |  |  |
| 1 | (peer* adj1 (program* or specialist* or support* or certifi* or support-specialist* or report or decision* or experienc* or intervent* or social support or community servic* or health or coach* or recovery-coach*or led or lead* or deliver* or run* or held or direct* or online)).mp. | 10653 |
| 2 | peer*.mp. adj1 Social Support/ | 6446 |
| 3 | (share* adj1 (decisionmak* or support* or communit*)).mp. | 944 |
| 4 | share.mp. adj1 (decision making.mp. or Decision Making/) [mp=title, book title, abstract, original title, name of substance word, subject heading word, floating sub-heading word, keyword heading word, organism supplementary concept word, protocol supplementary concept word, rare disease supplementary concept word, unique identifier, synonyms, population supplementary concept word, anatomy supplementary concept word] | 167 |
| 5 | (Youtube or facebook* or smartphone* or mHealth or eHealth or website* or GPS or social-media* or app* or telemet* or telemed* or telepsychiat* or telehealth or telecare* or telemental-health* or ehealth or mhealth or mobile* or mobile-health* or mobile-technolog* or mobilephone* or cellular-phone* or cellphon* or textmessag* or text-messag* or short-message-service* or SMS or internet-health* or internet* or online* or social-media* or tablet* or bluetooth* or avatar* or GPS or global-positioning-devic* or globalpositioning-system* or geographical-information-system* or telecommunic* or virtual-real* or VR or website* or web-base* or web-brows* or remoteconsult* or remote-sens* or artificial-intelligenc* or AI or game-theor* or user-computer-interfac* or computer-simulat* or speech-recognition-softwar* or computer-assisted-therap* or computer-base* or wirelesstechnolog* or remote-sensing-technolog* or informatics-applic* or handheldcomputer*).mp. | 7870209 |
| 6 | Geographic Information Systems/ | 10117 |
| 7 | Telecommunications/ | 5079 |
| 8 | Virtual Reality/ | 8364 |
| 9 | Virtual Reality Exposure Therapy/ | 1150 |
| 10 | Medical Informatics Applications/ | 2554 |
| 11 | Telemedicine/ | 44726 |
| 12 | Text Messaging/ | 5114 |
| 13 | Social Media/ | 19829 |
| 14 | User-Computer Interface/ | 40508 |
| 15 | Medical Informatics Applications/ | 2554 |
| 16 | Computer Simulation/ | 222815 |
| 17 | Web Browser/ | 1418 |
| 18 | Telemetry/ | 10401 |
| 19 | Game Theory/ | 4276 |
| 20 | Smartphone/ | 11642 |
| 21 | Computers, Handheld/ | 4227 |
| 22 | (computer* adj1 palm-top*).mp. [mp=title, book title, abstract, original title, name of substance word, subject heading word, floating sub-heading word, keyword heading word, organism supplementary concept word, protocol supplementary concept word, rare disease supplementary concept word, unique identifier, synonyms, population supplementary concept word, anatomy supplementary concept word] | 39 |
| 23 | (PDA or PC or personal-comput*).mp. [mp=title, book title, abstract, original title, name of substance word, subject heading word, floating sub-heading word, keyword heading word, organism supplementary concept word, protocol supplementary concept word, rare disease supplementary concept word, unique identifier, synonyms, population supplementary concept word, anatomy supplementary concept word] | 94225 |
| 24 | (personal* adj1 digital-assist*).mp. [mp=title, book title, abstract, original title, name of substance word, subject heading word, floating sub-heading word, keyword heading word, organism supplementary concept word, protocol supplementary concept word, rare disease supplementary concept word, unique identifier, synonyms, population supplementary concept word, anatomy supplementary concept word] | 950 |
| 25 | (tablet* adj1 computer*).mp. [mp=title, book title, abstract, original title, name of substance word, subject heading word, floating sub-heading word, keyword heading word, organism supplementary concept word, protocol supplementary concept word, rare disease supplementary concept word, unique identifier, synonyms, population supplementary concept word, anatomy supplementary concept word] | 1105 |
| 26 | Microcomputers/ | 14408 |
| 27 | Artificial Intelligence/ | 55256 |
| 28 | Mobile Applications/ | 14969 |
| 29 | Online Systems/ | 8579 |
| 30 | Cell Phone/ | 10499 |
| 31 | Therapy, Computer-Assisted/ | 7044 |
| 32 | Internet/ or Internet-Based Intervention/ | 87301 |
| 33 | Software/ | 132908 |
| 34 | Social Networking/ | 6046 |
| 35 | Blogging/ | 1109 |
| 36 | Robotics/ | 30687 |
| 37 | or/1-4 | 15701 |
| 38 | or/5-36 | 8004691 |
| 39 | 37 and 38 | 7931 |
| 40 | (SMI or Serious-mental-illness* or serious-mental-disease* or Severe-mental-illness* or severely-mentally-ill* or persistent-mental-illness* or chronic-mental-illness* or chronically-mentally-ill* or schizophren* or schizoaffective* or Schizoid* or Bipolar-Affective* or Bipolar-disorder* or Bipolar-depression* or Bipolar-illness* or Paranoia* or Paranoid* or psychos* or psychot* or mania* or Manic* or bipola* or PTSD or post-trauma* or posttrauma*).mp. [mp=title, book title, abstract, original title, name of substance word, subject heading word, floating sub-heading word, keyword heading word, organism supplementary concept word, protocol supplementary concept word, rare disease supplementary concept word, unique identifier, synonyms, population supplementary concept word, anatomy supplementary concept word] | 639724 |
| 41 | Schizophrenia Spectrum and Other Psychotic Disorders/ | 3 |
| 42 | Bipolar Disorder/ | 47425 |
| 43 | Schizoid Personality Disorder/ | 606 |
| 44 | personality disorder.mp. or Personality Disorders/ | 47061 |
| 45 | Stress Disorders, Post-Traumatic/ | 45744 |
| 46 | Mental Disorders/ or mental disorder*.mp. | 228802 |
| 47 | (mental* adj1 (ill* or distress*)).mp. | 48079 |
| 48 | mental illness*.mp. | 38308 |
| 49 | Anxiety Disorders/ | 44884 |
| 50 | Mood Disorders/ | 16594 |
| 51 | (psychiatric disorder* or affective disorder* or depress* or anxi* or dysthymic disorder* or persistent depressive disorder* or neurotic disorder* or seasonal affective disorder* or mood or panic* or agoraphob* or phobi* or social anxi* or general* anxi* or obsess* compuls* or impulse control disorder* or somatoform or sleep disorder* or sleep disturbance* or sleep problem* or psychotrauma* or traumatic or self-harm or suicid* or automutilation or self-injurious behavio*).mp. [mp=title, book title, abstract, original title, name of substance word, subject heading word, floating sub-heading word, keyword heading word, organism supplementary concept word, protocol supplementary concept word, rare disease supplementary concept word, unique identifier, synonyms, population supplementary concept word, anatomy supplementary concept word] | 1223389 |
| 52 | (eating disorder*OR binge-eating or anorexia or bulimia or bulimi*).mp. [mp=title, book title, abstract, original title, name of substance word, subject heading word, floating sub-heading word, keyword heading word, organism supplementary concept word, protocol supplementary concept word, rare disease supplementary concept word, unique identifier, synonyms, population supplementary concept word, anatomy supplementary concept word] | 43359 |
| 53 | (evaluation or intervention or trial or rando* or control* study protocol or pilot or clinical protocol or RCT* or feasibility or before after or pre post or cluster or quasiexperimental).mp. | 4764690 |
| 54 | Clinical Trials as Topic/ or Randomized Controlled Trials as Topic/ | 383819 |
| 55 | or/40-52 | 1774009 |
| 56 | 53 or 54 | 4848071 |
| 57 | 39 and 55 and 56 | 1242 |

| Embase <1980 to 2025 Week 24> | | |
| --- | --- | --- |
|  |  |  |
| 1 | (peer* adj1 (program* or specialist* or support* or certifi* or support-specialist* or report or decision* or experienc* or intervent* or social support or community servic* or health or coach* or recovery-coach*or led or lead* or deliver* or run* or held or direct* or online)).mp. | 17473 |
| 2 | peer*.mp. adj1 Social Support/ | 8872 |
| 3 | (share* adj1 (decisionmak* or support* or communit*)).mp. | 1925 |
| 4 | share.mp. adj1 (decision making.mp. or Decision Making/) [mp=title, abstract, heading word, drug trade name, original title, device manufacturer, drug manufacturer, device trade name, keyword heading word, floating subheading word, candidate term word] | 324 |
| 5 | (Youtube or facebook* or smartphone* or mHealth or eHealth or website* or GPS or social-media* or app* or telemet* or telemed* or telepsychiat* or telehealth or telecare* or telemental-health* or ehealth or mhealth or mobile* or mobile-health* or mobile-technolog* or mobilephone* or cellular-phone* or cellphon* or textmessag* or text-messag* or short-message-service* or SMS or internet-health* or internet* or online* or social-media* or tablet* or bluetooth* or avatar* or GPS or global-positioning-devic* or globalpositioning-system* or geographical-information-system* or telecommunic* or virtual-real* or VR or website* or web-base* or web-brows* or remoteconsult* or remote-sens* or artificial-intelligenc* or AI or game-theor* or user-computer-interfac* or computer-simulat* or speech-recognition-softwar* or computer-assisted-therap* or computer-base* or wirelesstechnolog* or remote-sensing-technolog* or informatics-applic* or handheldcomputer*).mp. | 11932162 |
| 6 | Geographic Information Systems/ | 14772 |
| 7 | Telecommunications/ | 30071 |
| 8 | Virtual Reality/ | 33113 |
| 9 | Virtual Reality Exposure Therapy/ | 1188 |
| 10 | Medical Informatics Applications/ | 24736 |
| 11 | Telemedicine/ | 53082 |
| 12 | Text Messaging/ | 9343 |
| 13 | Social Media/ | 65061 |
| 14 | User-Computer Interface/ | 38058 |
| 15 | Medical Informatics Applications/ | 24736 |
| 16 | Computer Simulation/ | 147086 |
| 17 | Web Browser/ | 8642 |
| 18 | Telemetry/ | 21619 |
| 19 | Game Theory/ | 10437 |
| 20 | Smartphone/ | 34294 |
| 21 | Computers, Handheld/ | 1963 |
| 22 | (computer* adj1 palm-top*).mp. [mp=title, abstract, heading word, drug trade name, original title, device manufacturer, drug manufacturer, device trade name, keyword heading word, floating subheading word, candidate term word] | 42 |
| 23 | (PDA or PC or personal-comput*).mp. [mp=title, abstract, heading word, drug trade name, original title, device manufacturer, drug manufacturer, device trade name, keyword heading word, floating subheading word, candidate term word] | 1462721 |
| 24 | (personal* adj1 digital-assist*).mp. [mp=title, abstract, heading word, drug trade name, original title, device manufacturer, drug manufacturer, device trade name, keyword heading word, floating subheading word, candidate term word] | 2649 |
| 25 | (tablet* adj1 computer*).mp. [mp=title, abstract, heading word, drug trade name, original title, device manufacturer, drug manufacturer, device trade name, keyword heading word, floating subheading word, candidate term word] | 5875 |
| 26 | Microcomputers/ | 14936 |
| 27 | Artificial Intelligence/ | 104756 |
| 28 | Mobile Applications/ | 26959 |
| 29 | Online Systems/ | 32897 |
| 30 | Cell Phone/ | 24810 |
| 31 | Therapy, Computer-Assisted/ | 4914 |
| 32 | Internet/ or Internet-Based Intervention/ | 134497 |
| 33 | Software/ | 315262 |
| 34 | Social Networking/ | 28345 |
| 35 | Blogging/ | 1060 |
| 36 | Robotics/ | 50754 |
| 37 | or/1-4 | 25669 |
| 38 | or/5-36 | 13203425 |
| 39 | 37 and 38 | 15011 |
| 40 | (SMI or Serious-mental-illness* or serious-mental-disease* or Severe-mental-illness* or severely-mentally-ill* or persistent-mental-illness* or chronic-mental-illness* or chronically-mentally-ill* or schizophren* or schizoaffective* or Schizoid* or Bipolar-Affective* or Bipolar-disorder* or Bipolar-depression* or Bipolar-illness* or Paranoia* or Paranoid* or psychos* or psychot* or mania* or Manic* or bipola* or PTSD or post-trauma* or posttrauma*).mp. [mp=title, abstract, heading word, drug trade name, original title, device manufacturer, drug manufacturer, device trade name, keyword heading word, floating subheading word, candidate term word] | 989205 |
| 41 | Schizophrenia Spectrum and Other Psychotic Disorders/ | 114668 |
| 42 | Bipolar Disorder/ | 83824 |
| 43 | Schizoid Personality Disorder/ | 2844 |
| 44 | personality disorder.mp. or Personality Disorders/ | 60299 |
| 45 | Stress Disorders, Post-Traumatic/ | 92562 |
|  |  |  |
|  |  |  |
| 46 | Mental Disorders/ or mental disorder*.mp. | 365428 |
| 47 | (mental* adj1 (ill* or distress*)).mp. | 72517 |
| 48 | mental illness*.mp. | 60118 |
| 49 | Anxiety Disorders/ | 113139 |
| 50 | Mood Disorders/ | 59209 |
| 51 | (psychiatric disorder* or affective disorder* or depress* or anxi* or dysthymic disorder* or persistent depressive disorder* or neurotic disorder* or seasonal affective disorder* or mood or panic* or agoraphob* or phobi* or social anxi* or general* anxi* or obsess* compuls* or impulse control disorder* or somatoform or sleep disorder* or sleep disturbance* or sleep problem* or psychotrauma* or traumatic or self-harm or suicid* or automutilation or self-injurious behavio*).mp. [mp=title, abstract, heading word, drug trade name, original title, device manufacturer, drug manufacturer, device trade name, keyword heading word, floating subheading word, candidate term word] | 1941754 |
| 52 | (eating disorder*OR binge-eating or anorexia or bulimia or bulimi*).mp. [mp=title, abstract, heading word, drug trade name, original title, device manufacturer, drug manufacturer, device trade name, keyword heading word, floating subheading word, candidate term word] | 120678 |
| 53 | (evaluation or intervention or trial or rando* or control* study protocol or pilot or clinical protocol or RCT* or feasibility or before after or pre post or cluster or quasiexperimental).mp. | 7981672 |
| 54 | Clinical Trials as Topic/ or Randomized Controlled Trials as Topic/ | 418174 |
| 55 | or/40-52 | 2743952 |
| 56 | 53 or 54 | 7981672 |
| 57 | 39 and 55 and 56 | 2629 |
| 58 | limit 57 to (article or article in press) | 1583 |

| EBM Reviews - Cochrane Central Register of Controlled Trials <May 2025> | | |
| --- | --- | --- |
|  |  |  |
| 1 | (peer* adj1 (program* or specialist* or support* or certifi* or support-specialist* or report or decision* or experienc* or intervent* or social support or community servic* or health or coach* or recovery-coach*or led or lead* or deliver* or run* or held or direct* or online)).mp. | 3676 |
| 2 | peer*.mp. adj1 Social Support/ | 546 |
| 3 | (share* adj1 (decisionmak* or support* or communit*)).mp. | 163 |
| 4 | share.mp. adj1 (decision making.mp. or Decision Making/) [mp=title, original title, abstract, floating sub-heading word, mesh headings, heading words, keyword] | 23 |
| 5 | (Youtube or facebook* or smartphone* or mHealth or eHealth or website* or GPS or social-media* or app* or telemet* or telemed* or telepsychiat* or telehealth or telecare* or telemental-health* or ehealth or mhealth or mobile* or mobile-health* or mobile-technolog* or mobilephone* or cellular-phone* or cellphon* or textmessag* or text-messag* or short-message-service* or SMS or internet-health* or internet* or online* or social-media* or tablet* or bluetooth* or avatar* or GPS or global-positioning-devic* or globalpositioning-system* or geographical-information-system* or telecommunic* or virtual-real* or VR or website* or web-base* or web-brows* or remoteconsult* or remote-sens* or artificial-intelligenc* or AI or game-theor* or user-computer-interfac* or computer-simulat* or speech-recognition-softwar* or computer-assisted-therap* or computer-base* or wirelesstechnolog* or remote-sensing-technolog* or informatics-applic* or handheldcomputer*).mp. | 662209 |
| 6 | Geographic Information Systems/ | 85 |
| 7 | Telecommunications/ | 112 |
| 8 | Virtual Reality/ | 1423 |
| 9 | Virtual Reality Exposure Therapy/ | 381 |
| 10 | Medical Informatics Applications/ | 43 |
| 11 | Telemedicine/ | 4506 |
| 12 | Text Messaging/ | 1721 |
| 13 | Social Media/ | 640 |
| 14 | User-Computer Interface/ | 1509 |
| 15 | Medical Informatics Applications/ | 43 |
| 16 | Computer Simulation/ | 2817 |
| 17 | Web Browser/ | 11 |
| 18 | Telemetry/ | 297 |
| 19 | Game Theory/ | 40 |
| 20 | Smartphone/ | 1276 |
| 21 | Computers, Handheld/ | 391 |
| 22 | (computer* adj1 palm-top*).mp. [mp=title, original title, abstract, floating sub-heading word, mesh headings, heading words, keyword] | 9 |
| 23 | (PDA or PC or personal-comput*).mp. [mp=title, original title, abstract, floating sub-heading word, mesh headings, heading words, keyword] | 18931 |
| 24 | (personal* adj1 digital-assist*).mp. [mp=title, original title, abstract, floating sub-heading word, mesh headings, heading words, keyword] | 177 |
| 25 | (tablet* adj1 computer*).mp. [mp=title, original title, abstract, floating sub-heading word, mesh headings, heading words, keyword] | 978 |
| 26 | Microcomputers/ | 286 |
| 27 | Artificial Intelligence/ | 790 |
| 28 | Mobile Applications/ | 2417 |
| 29 | Online Systems/ | 193 |
| 30 | Cell Phone/ | 1018 |
| 31 | Therapy, Computer-Assisted/ | 1541 |
| 32 | Internet/ or Internet-Based Intervention/ | 6213 |
| 33 | Software/ | 1453 |
| 34 | Social Networking/ | 226 |
| 35 | Blogging/ | 24 |
| 36 | Robotics/ | 1206 |
| 37 | or/1-4 | 3970 |
| 38 | or/5-36 | 676574 |
| 39 | 37 and 38 | 2322 |
| 40 | (SMI or Serious-mental-illness* or serious-mental-disease* or Severe-mental-illness* or severely-mentally-ill* or persistent-mental-illness* or chronic-mental-illness* or chronically-mentally-ill* or schizophren* or schizoaffective* or Schizoid* or Bipolar-Affective* or Bipolar-disorder* or Bipolar-depression* or Bipolar-illness* or Paranoia* or Paranoid* or psychos* or psychot* or mania* or Manic* or bipola* or PTSD or post-trauma* or posttrauma*).mp. [mp=title, original title, abstract, floating sub-heading word, mesh headings, heading words, keyword] | 89071 |
| 41 | Schizophrenia Spectrum and Other Psychotic Disorders/ | 39 |
| 42 | Bipolar Disorder/ | 3596 |
| 43 | Schizoid Personality Disorder/ | 9 |
| 44 | personality disorder.mp. or Personality Disorders/ | 3196 |
| 45 | Stress Disorders, Post-Traumatic/ | 4349 |
| 46 | Mental Disorders/ or mental disorder*.mp. | 14586 |
| 47 | (mental* adj1 (ill* or distress*)).mp. | 6692 |
| 48 | mental illness*.mp. | 5890 |
| 49 | Anxiety Disorders/ | 6495 |
| 50 | Mood Disorders/ | 1155 |
| 51 | (psychiatric disorder* or affective disorder* or depress* or anxi* or dysthymic disorder* or persistent depressive disorder* or neurotic disorder* or seasonal affective disorder* or mood or panic* or agoraphob* or phobi* or social anxi* or general* anxi* or obsess* compuls* or impulse control disorder* or somatoform or sleep disorder* or sleep disturbance* or sleep problem* or psychotrauma* or traumatic or self-harm or suicid* or automutilation or self-injurious behavio*).mp. [mp=title, original title, abstract, floating sub-heading word, mesh headings, heading words, keyword] | 211628 |
| 52 | (eating disorder*OR binge-eating or anorexia or bulimia or bulimi*).mp. [mp=title, original title, abstract, floating sub-heading word, mesh headings, heading words, keyword] | 7824 |
| 53 | or/40-52 | 269702 |
| 54 | 39 and 53 | 934 |

| APA PsycInfo <1806 to June 2025 Week 2> | | |
| --- | --- | --- |
|  |  |  |
| 1 | (peer* adj1 (program* or specialist* or support* or certifi* or support-specialist* or report or decision* or experienc* or intervent* or social support or community servic* or health or coach* or recovery-coach*or led or lead* or deliver* or run* or held or direct* or online)).mp. | 13182 |
| 2 | peer*.mp. adj1 Social Support/ | 5788 |
| 3 | (share* adj1 (decisionmak* or support* or communit*)).mp. | 632 |
| 4 | share.mp. adj1 (decision making.mp. or Decision Making/) [mp=title, abstract, heading word, table of contents, key concepts, original title, tests & measures, mesh word] | 200 |
| 5 | (Youtube or facebook* or smartphone* or mHealth or eHealth or website* or GPS or social-media* or app* or telemet* or telemed* or telepsychiat* or telehealth or telecare* or telemental-health* or ehealth or mhealth or mobile* or mobile-health* or mobile-technolog* or mobilephone* or cellular-phone* or cellphon* or textmessag* or text-messag* or short-message-service* or SMS or internet-health* or internet* or online* or social-media* or tablet* or bluetooth* or avatar* or GPS or global-positioning-devic* or globalpositioning-system* or geographical-information-system* or telecommunic* or virtual-real* or VR or website* or web-base* or web-brows* or remoteconsult* or remote-sens* or artificial-intelligenc* or AI or game-theor* or user-computer-interfac* or computer-simulat* or speech-recognition-softwar* or computer-assisted-therap* or computer-base* or wirelesstechnolog* or remote-sensing-technolog* or informatics-applic* or handheldcomputer*).mp. | 2046205 |
| 6 | exp Information Systems/ | 88254 |
| 7 | Telecommunications/ | 1628 |
| 8 | Virtual Reality/ | 13574 |
| 9 | Virtual Reality Exposure Therapy/ | 366 |
| 10 | exp "Information and Communication Technology"/ | 286084 |
| 11 | Telemedicine/ | 10500 |
| 12 | Text Messaging/ | 1863 |
| 13 | Social Media/ | 21705 |
| 14 | exp Human Computer Interaction/ | 31322 |
| 15 | exp Decision Support Systems/ | 4003 |
| 16 | Computer Simulation/ | 5036 |
| 17 | exp Human Computer Interaction/ | 31322 |
| 18 | Telemetry/ | 215 |
| 19 | Game Theory/ | 3855 |
| 20 | exp Smartphones/ | 4090 |
| 21 | exp Tablet Computers/ or exp Computer Applications/ | 99642 |
| 22 | (computer* adj1 palm-top*).mp. [mp=title, abstract, heading word, table of contents, key concepts, original title, tests & measures, mesh word] | 32 |
| 23 | (PDA or PC or personal-comput*).mp. [mp=title, abstract, heading word, table of contents, key concepts, original title, tests & measures, mesh word] | 8665 |
| 24 | (personal* adj1 digital-assist*).mp. [mp=title, abstract, heading word, table of contents, key concepts, original title, tests & measures, mesh word] | 477 |
| 25 | (tablet* adj1 computer*).mp. [mp=title, abstract, heading word, table of contents, key concepts, original title, tests & measures, mesh word] | 1758 |
| 26 | Microcomputers/ | 1353 |
| 27 | Artificial Intelligence/ | 14736 |
| 28 | Mobile Applications/ | 3572 |
| 29 | exp Computer Assisted Instruction/ | 22172 |
| 30 | Cell Phone/ | 5939 |
| 31 | exp Computer Assisted Therapy/ | 18332 |
| 32 | Internet/ or Internet-Based Intervention/ | 32234 |
| 33 | exp Computer Software/ or exp Computer Applications/ | 98627 |
| 34 | exp Online Social Networks/ | 10908 |
| 35 | exp Social Media/ or exp Blog/ or exp Computer Mediated Communication/ | 51959 |
| 36 | Robotics/ | 8615 |
| 37 | or/1-4 | 17294 |
| 38 | or/5-36 | 2112216 |
| 39 | 37 and 38 | 7989 |
| 40 | (SMI or Serious-mental-illness* or serious-mental-disease* or Severe-mental-illness* or severely-mentally-ill* or persistent-mental-illness* or chronic-mental-illness* or chronically-mentally-ill* or schizophren* or schizoaffective* or Schizoid* or Bipolar-Affective* or Bipolar-disorder* or Bipolar-depression* or Bipolar-illness* or Paranoia* or Paranoid* or psychos* or psychot* or mania* or Manic* or bipola* or PTSD or post-trauma* or posttrauma*).mp. [mp=title, abstract, heading word, table of contents, key concepts, original title, tests & measures, mesh word] | 737886 |
| 41 | exp Schizophrenia/ or exp Psychosis/ | 136873 |
| 42 | Bipolar Disorder/ | 32826 |
| 43 | Schizoid Personality Disorder/ | 696 |
| 44 | personality disorder.mp. or Personality Disorders/ | 48773 |
| 45 | exp Posttraumatic Stress Disorder/ | 44934 |
| 46 | Mental Disorders/ or mental disorder*.mp. | 208950 |
| 47 | (mental* adj1 (ill* or distress*)).mp. | 77969 |
| 48 | mental illness*.mp. | 59713 |
| 49 | Anxiety Disorders/ | 23213 |
| 50 | Mood Disorders/ | 17064 |
| 51 | (psychiatric disorder* or affective disorder* or depress* or anxi* or dysthymic disorder* or persistent depressive disorder* or neurotic disorder* or seasonal affective disorder* or mood or panic* or agoraphob* or phobi* or social anxi* or general* anxi* or obsess* compuls* or impulse control disorder* or somatoform or sleep disorder* or sleep disturbance* or sleep problem* or psychotrauma* or traumatic or self-harm or suicid* or automutilation or self-injurious behavio*).mp. [mp=title, abstract, heading word, table of contents, key concepts, original title, tests & measures, mesh word] | 892281 |
| 52 | (eating disorder*OR binge-eating or anorexia or bulimia or bulimi*).mp. [mp=title, abstract, heading word, table of contents, key concepts, original title, tests & measures, mesh word] | 27944 |
| 53 | (evaluation or intervention or trial or rando* or control* study protocol or pilot or clinical protocol or RCT* or feasibility or before after or pre post or cluster or quasiexperimental).mp. | 1029147 |
| 54 | exp Randomized Clinical Trials/ or exp Clinical Trials/ or exp Randomized Controlled Trials/ | 14291 |
| 55 | or/40-52 | 1472391 |
| 56 | 53 or 54 | 1031172 |
| 57 | 39 and 55 and 56 | 1222 |
| 58 | limit 57 to (article or article in press) | 1583 |

**e-Supplement 3: Reference list of included studies**

1. Alvarez Jimenez M, Koval P, Schmaal L, Bendall S, O’Sullivan S, Cagliarini D, D’Alfonso S, Rice S, Valentine L, Penn DL, Miles C, Russon P, Phillips J, McEnery C, Lederman R, Killackey E, Mihalopoulos C, Gonzalez Blanch C, Gilbertson T, Lal S, Cotton SM, Herrman H, McGorry PD, Gleeson JFM. The Horyzons project: a randomized controlled trial of a novel online social therapy to maintain treatment effects from specialist first episode psychosis services. World Psychiatry. 2021;20:233–243. <https://doi.org/10.1002/wps.20858>
2. Bautista CL, Ralston AL, Brock RL, Hope DA. Peer coach support in internet based cognitive behavioral therapy for college students with social anxiety disorder: efficacy and acceptability. Cogent Psychology. 2022;9:2040160. <https://doi.org/10.1080/23311908.2022.2040160>
3. Ellis LA, Campbell AJ, Sethi S, O’Dea BM. Comparative randomized trial of an online cognitive behavioral therapy program and an online support group for depression and anxiety. Journal of CyberTherapy & Rehabilitation. 2011;4(4):461–467.
4. Finnerty MT, Layman DM, Chen Q, Leckman Westin E, Bermeo N, Ng Mak DS, Rajagopalan K, Hoagwood KE. Use of a web-based shared decision-making program: impact on ongoing treatment engagement and antipsychotic adherence. Psychiatric Services. 2018;69:1215–1221. <https://doi.org/10.1176/appi.ps.201800130>
5. Gumley AI, Bradstreet S, Ainsworth J, Allan S, Alvarez Jimenez M, Birchwood M, Briggs A, Bucci S, Cotton S, Engel L, French P, Lederman R, Lewis S, Machin M, MacLennan G, McLeod H, McMeekin N, Mihalopoulos C, Morton E, Norrie J, Reilly F, Schwannauer M, Singh SP, Sundram S, Thompson A, Williams C, Yung A, Aucott L, Farhall J, Gleeson J. Digital smartphone intervention to recognise and manage early warning signs in schizophrenia to prevent relapse: the EMPOWER feasibility cluster RCT. Health Technology Assessment. 2022;26(27). <https://doi.org/10.3310/HLZE0479>
6. Hamblen JL, Grubaugh AL, Davidson TM, Borkman AL, Bunnell BE, Ruggiero KJ. An online peer educational campaign to reduce stigma and improve help seeking in veterans with posttraumatic stress disorder. Telemedicine and e-Health. 2018;24:372–380. <https://doi.org/10.1089/tmj.2017.0305>
7. Henderson K, Reihm J, Koshal K, Wijangco J, Sara N, Miller N, Doyle M, Mallory A, Sheridan J, Guo CY, Oommen L, Rankin KP, Sanders S, Feinstein A, Mangurian C, Bove R. A closed loop digital health tool to improve depression care in multiple sclerosis: iterative design and cross sectional pilot randomized controlled trial and its impact on depression care. JMIR Formative Research. 2024;8:e52809. <https://doi.org/10.2196/52809>
8. Hensel JM, Shaw J, Ivers NM, Desveaux L, Vigod SN, Bouck Z, Onabajo N, Agarwal P, Mukerji G, Yang R, Nguyen M, Jeffs L, Jamieson T, Bhatia RS. Extending access to a web-based mental health intervention: who wants more, what happens to use over time, and is it helpful? Results of a concealed, randomized controlled extension study. BMC Psychiatry. 2019;19:39. <https://doi.org/10.1186/s12888-019-2030-x>
9. Kaplan K, Salzer MS, Solomon P, Brusilovskiy E, Cousounis P. Internet peer support for individuals with psychiatric disabilities: a randomized controlled trial. Social Science & Medicine. 2011;72:54–62. <https://doi.org/10.1016/j.socscimed.2010.09.037>
10. Kaveladze B, Shkel J, Le S, Marcotte V, Rushton K, Nguyen T, Schueller SM. Crowdsourcing integrated into a digital mental health platform for anxiety and depression: a pilot randomized controlled trial. Internet Interventions. 2024;38:100774. <https://doi.org/10.1016/j.invent.2024.100774>
11. Kelly EL, Braslow JT, Brekke JS. Using electronic health records to enhance a peer health navigator intervention: a randomized pilot test for individuals with serious mental illness and housing instability. Community Mental Health Journal. 2018;54:1172–1179. <https://doi.org/10.1007/s10597-018-0282-4>
12. Merza D, Amani B, Savoy C, Babiy Z, Bieling PJ, Streiner DL, Ferro MA, Van Lieshout RJ. Online peer delivered group cognitive behavioral therapy for postpartum depression: a randomized controlled trial. Acta Psychiatrica Scandinavica. 2024;150(5):422–432. <https://doi.org/10.1111/acps.13611>
13. Morriss R, Kaylor Hughes C, Rawsthorne M, Coulson N, Simpson S, Guo B, James M, Lathe J, Moran P, Tata LJ, Williams L. A direct to public peer support program (Big White Wall) versus web based information to aid the self management of depression and anxiety: results and challenges of an automated randomized controlled trial. Journal of Medical Internet Research. 2021;23(4):e23487. <https://doi.org/10.2196/23487>
14. Ong LE, Speicher S, Villasenor D, Kim J, Jacobs A, Macia KS, Cloitre M. Brief peer-supported web-based skills training in affective and interpersonal regulation (BPS webSTAIR) for trauma-exposed veterans in the community: randomized controlled trial. Journal of Medical Internet Research. 2024;26:e52130. <https://doi.org/10.2196/52130>
15. Pfeiffer PN, Pope B, Houck M, Benn-Burton W, Zivin K, Ganoczy D, Kim HM, Walters H, Emerson L, Nelson CB, Abraham KM, Valenstein M. Effectiveness of peer-supported computer-based CBT for depression among veterans in primary care. Psychiatric Services. 2020;71:256–262. <https://doi.org/10.1176/appi.ps.201900283>
16. Possemato K, Johnson EM, Emery JB, Wade M, Acosta MC, Marsch LA, Rosenblum A, Maisto SA. A pilot study comparing peer-supported web-based CBT to self-managed web CBT for primary care veterans with PTSD and hazardous alcohol use. Psychiatric Rehabilitation Journal. 2019;42:305–313. <https://doi.org/10.1037/prj0000334>
17. Proudfoot J, Parker G, Manicavasagar V, Hadzi-Pavlovic D, Whitton A, Nicholas J, Smith M, Burckhardt R. Effects of adjunctive peer support on perceptions of illness control and understanding in an online psychoeducation program for bipolar disorder: a randomised controlled trial. J Affect Disord. 2012;142:98–105. <https://doi.org/10.1016/j.jad.2012.05.015>
18. Sawyer A, Kaim A, Le H-N, McDonald D, Mittinty M, Lynch J, Sawyer M. The effectiveness of an app-based nurse-moderated program for new mothers with depression and parenting problems (eMums Plus): pragmatic randomized controlled trial. J Med Internet Res. 2019;21:e13689. <https://doi.org/10.2196/13689>
19. Schlosser DA, Campellone TR, Truong B, Etter K, Vergani S, Komaiko K, Vinogradov S. Efficacy of PRIME, a mobile app intervention designed to improve motivation in young people with schizophrenia. Schizophr Bull. 2018;44:1010–1020. <https://doi.org/10.1093/schbul/sby078>
20. Schulz A, Stolz T, Vincent A, Krieger T, Andersson G, Berger T. A sorrow shared is a sorrow halved? A three-arm randomized controlled trial comparing internet-based clinician-guided individual versus group treatment for social anxiety disorder. Behav Res Ther. 2016;84:14–26. <https://doi.org/10.1016/j.brat.2016.07.001>
21. Shalaby R, Spurvey P, Knox M, Rathwell R, Vuong W, Surood S, Urichuk L, Snaterse M, Greenshaw AJ, Li X, Agyapong VIO. Clinical outcomes in routine evaluation measures for patients discharged from acute psychiatric care: four arm peer and text messaging support controlled observational study. Int J Environ Res Public Health. 2022;19(7):3798. <https://doi.org/10.3390/ijerph19073798>
22. Shorey S, Chee CYI, Ng ED, Lau Y, Dennis C L, Chan YH. Evaluation of a technology-based peer-support intervention program for preventing postnatal depression (Part 1): randomized controlled trial. J Med Internet Res. 2019;21(8):e12410. <https://doi.org/10.2196/12410>
23. Simmons M, Batchelor S, Dimopoulos-Bick T, Howe D. The Choice Project: peer workers promoting shared decision making at a youth mental health service. Psychiatric Services. 2017;68:764–770. <https://doi.org/10.1176/appi.ps.201600388>
24. Simon D, Kriston L, von Wolff A, Buchholz A, Vietor C, Hecke T, et al. Effectiveness of a web-based, individually tailored decision aid for depression or acute low back pain: a randomized controlled trial. Patient Educ Couns. 2012;87:360–368. <https://doi.org/10.1016/j.pec.2011.10.009>
25. Tomasino KN, Lattie EG, Ho J, Palac HL, Kaiser SM, Mohr DC. Harnessing peer support in an online intervention for older adults with depression. Am J Geriatr Psychiatry. 2017;25:1109–1119. <https://doi.org/10.1016/j.jagp.2017.04.015>
26. Ugarte DA, Cumberland WG, Singh P, Saadat S, Garett R, Young SD. A HOPE online community peer support intervention for help seeking: a randomized controlled trial. Psychiatric Services. 2023;74:648–651. <https://doi.org/10.1176/appi.ps.202000817>
27. Westerhof GJ, Lamers SMA, Postel MG, Bohlmeijer ET. Online therapy for depressive symptoms: an evaluation of counselor-led and peer-supported life review therapy. The Gerontologist. 2019;59:135–146. <https://doi.org/10.1093/geront/gnx140>
28. Kordy H, Wolf M, Aulich K, Bürgy M, Hegerl U, Hüsing J, Puschner B, Rummel-Kluge C, Vedder H, Backenstrass M. Internet-delivered disease management for recurrent depression: a multicenter randomized controlled trial. Psychotherapy and Psychosomatics. 2016;85:91–98. <https://doi.org/10.1159/000441951>
29. Yamaguchi S, Taneda A, Matsunaga A, Sasaki N, Mizuno M, Sawada Y, Sakata M, Fukui S, Hisanaga F, Bernick P, Ito J. Efficacy of a peer-led, recovery-oriented shared decision-making system: a pilot randomized controlled trial. Psychiatric Services. 2017;68:1307–1311. <https://doi.org/10.1176/appi.ps.2016005>

**eTable 1: Study and population characteristics**

| **Study** | **Country** | **Study Setting (s)** | **Design** | **Sample size** | **Age (mean, SD)** | **Gender (% male)** | **Primary condition(s)** |
| --- | --- | --- | --- | --- | --- | --- | --- |
| **Alvarez-Jimenez 2021** | Australia | Specialised community clinical setting | RCT | 170 | 20.9, 2.9 | 52.9 | Affective and non affective psychosis |
| **Bautista 2022** | USA | Community | RCT | 35 | 21.9, 4.8 | 28.6 | Social anxiety |
| **Ellis 2011** | Australia | Community | RCT | 39 | 19.7, 1.7 | 23 | Depression (Low to moderate levels of psychological distress) |
| **Finnerty 2018** | USA | Primary care: mental health outpatient clinics in New York state | Non-randomized, propensity score–matched cohort study | 472 | 44, 12.3 (control) | 18.6 | Mixed conditions |
| **Gumley 2022** | UK and Australia | community mental health services | RCT | 73 | 43 | 51 | Schizophrenia related disorders |
| **Hamblen 2019** | USA | Veterans Association PTSD specialized outpatient clinic | RCT | 60 | 42.2, 12.6 | 70 | Posttraumatic stress disorder (PTSD) |
| **Hensel 2019** | Canada | Outpatient mental health programs affiliated with 3 hospital programs | RCT | 812 | 41.5, 13.4 | 27 | Mixed conditions |
| **Kaplan 2011** | USA | Community | RCT | 300 | 47, nr | 34 | Schizophrenia Spectrum Disorder, affective disorders |
| **Kaplan 2014/ O'Shea 2019** | USA | Community | RCT | 131 | 37, 7.7 | 0 | Schizophrenia Spectrum Disorder, or mood disorders |
| **Kaveladze 2024** | USA | Community - participants were recruited from Mental Health America's Screening to-Supports platform (S2S) online screening platform. | RCT | 107 | 34.6, 13.4 (control) | 38.3 | Depression or anxiety |
| **Kelly 2018** | USA | Community mental health services | RCT (pilot) | 20 | 49.3, 10.9 (control | 56% (control) | Mood disorder, schizophrenia |
| **Merza 2023** | Canada | Community | RCT | 183 | 31.6, 4.9 (control) | 0 | Post-natal depression |
| **Morriss 2021** | UK | Community | RCT | 790 | 38, 13.8 | 19 | Depression and Anxiety (mild-moderate) |
| **Ong 2024** | USA | Community | RCT | 178 | 48.1, 9.0 | 72 (control) | PTSD or depression, or both |
| **Pfeiffer 2020** | USA | Primary Care | RCT | 330 | 51.6, 14.9 | 80 | Depression |
| **Possemato 2019** | USA | Primary Care | Pilot RCT | 30 | 39.0, 9,0 | 93 | PTSD |
| **Proudfoot 2012** | Australia | Community | RCT | 407 | NR | 30.6 (control) | Bipolar disorder |
| **Sawyer 2019** | Australia | Community | RCT | 133 | 32.2, 4.0 (control) | 0 | Postnatal depression |
| **Schlosser 2018** | USA | Community | RCT | 43 | 23.8, 4.5 (control) | 65 (control) | Schizophrenia, schizoaffective disorders |
| **Schulz 2016** | Switzerland, Austria and Germany | Community | RCT | 149 | 35.4, 11.2 (control) | 47 | Social anxiety disorder |
| **Shalaby 2022** | Canada | Community | RCT | 181 | NR | 43.1 | Mood or psychotic disorder |
| **Shorey 2019** | Singapore | Outpatient tertiary hospital | RCT | 138 | 32.1, 4.4 | 0 | At risk of post-natal depression |
| **Simon 2011** | USA | Community- Primarily Internet-based and mental health clinics; | RCT | 118 | NR | 28 | Bipolar disorder |
| **Simmons 2017** | Australia | community - youth mental health service | Historical comparison group design | 229 | 18.4, 2.5 (control) | 44 (control) | Mixed conditions |
| **Tomasino 2017** | USA | Community | RCT + Feasibility | 47 | 69.6, 4.1 | 31.9 (control) | Depression |
| **Ugarte 2022** | USA | Community | RCT | 300 | 39.4, 12.4 | 18 (control) | Moderate to severe generalized anxiety disorder |
| **Westerhof 2019** | Netherlands | Community | RCT | 58 | 53.8, 8.4 | 22.4 | Light to moderate depressive symptoms |
| **Wolf 2011/Kordy 2016** | Germany | Outpatient psychiatric departments | RCT | 236 | 41.8, 10.4 | 37.2 | Recurrent major depressive disorder |
| **Yamaguchi 2017** | Japan | Outpatient psychiatric clinic and one psychiatric hospital) | RCT (pilot) | 56 | NR | NR | Schizophrenia (70%) another psychotic disorder patients |

eTable 2: Intervention characteristics

| **Study** | **Brief Description of the Intervention** | **Length & Co-Design** | **Safety & Costs** | **Control Group** | **Theory Based** | **Peer Support Type & Mode** | **Digital Therapeutic Format** |
| --- | --- | --- | --- | --- | --- | --- | --- |
| Alvarez-Jimenez 2021 | Horyzons: MOST platform with therapy modules, peer-to-peer social networking, peer moderation, and expert support | 18 months, co-designed with young people | Safety NR; costs lower than TAU (healthcare –AU$4789.59; societal –AU$5131.14; P<.001) | Treatment as usual post-discharge | MOST model; self-determination theory; IPS model | Peer-professional supported, online forums & peer networking | Online platform |
| Bautista 2022 | Self-guided internet CBT (6 modules) | 6 weeks, not co-designed | Safety NR; Costs NR | Waitlist | CBT | Peer-based intervention, online | Website |
| Ellis 2011 | MoodGYM CBT + MoodGarden online peer support | 3 weeks, not co-designed | Safety NR; Costs NR | No treatment, questionnaires | CBT | Peer-based, online forums/messages | Website |
| Finnerty 2018 | MyCHOIS/CommonsGround shared decision-making tool with peer involvement | N/A (3-year data) | Safety NR; Costs NR | Matched control group | NR | Peer-professional supported, web-based tool | Online platform |
| Gumley 2022 | EMPOWER app: monitoring and prompts with PPI | 4-week monitoring + 12-month follow-up, co-designed | Adverse events monitored; Costs NR | TAU secondary care | Cognitive interpersonal model | Peer-professional supported, app-based | Mobile app |
| Hamblen 2019 | AboutFace: web-based educational campaign with peer stories for veterans with PTSD | 2 weeks, co-designed | Safety NR; Costs NR | Usual care | Behaviour science | Peer-professional supported, online narratives | Website |
| Hensel 2019 | BWW: CBT & moderated web-based support | 3 months, not co-designed | 1 death unrelated; Costs NR | Delayed treatment 3 months | CBT | Peer-professional supported, online | Web-based platform |
| Kaplan 2011 | Internet Peer Support: listserv & bulletin board | 12 months, not co-designed | Safety NR; Costs NR | Waitlist | NR | Peer-professional supported, online | Email/listserv |
| Kaplan 2014 / O'Shea 2019 | 12 online parenting sessions + moderated listserv | 12 months, not co-designed | Safety NR; Costs NR | Enhanced usual care: monthly factsheets | NR | Peer-professional supported, online | Web-based + videos |
| Kaveladze 2024 | “Overcoming Thoughts” platform with crowdsourced peer support | 8 weeks + 16-week follow-up, not co-designed | Suicidality reported via PHQ-9; Costs NR | Platform without peer support | CBT | Peer-based intervention, online | Web platform |
| Kelly 2018 | C-PHR + Bridge: 1:1 peer health navigator + electronic health record | NR (6 months) | Safety NR; Costs NR | Waitlist | NR | Peer-based, blended | Face-to-face + electronic tool |
| Merza 2023 | Online peer-delivered CBT (weekly 2h sessions via Zoom) | 9 weeks, not co-designed | Safety NR; Costs NR | Waitlist | CBT | Peer-based, live group | Videoconferencing |
| Morriss 2021 | Big White Wall: assessment + moderated peer support + guided programs | 6 months, input from advisory panel | Safety NR; Some pre-intervention costs | Active control (NHS Moodzone) | CBT & social support | Peer-professional supported, online | Web-based platform |
| Ong 2024 | BPS webSTAIR: 6 modules + 8-week follow-up | 10 weeks + 8-week follow-up, not co-designed | Safety NR; Costs NR | Waitlist | Behaviour science | Peer-based, online modules | Web-based platform |
| Pfeiffer 2020 | Peer-supported computer-based CBT (Beating the Blues) | 3 months, not co-designed | Safety NR; Costs NR | EUC + UC | CBT | Peer-based, blended | Online + in-person/phone |
| Possemato 2019 | Thinking Forward: self-managed online modules with optional peer support | 12 weeks, not co-designed | Safety NR; Costs NR | Active control: self-managed CBT | CBT | Peer-based, blended | Online + in-person/phone |
| Proudfoot 2012 | Bipolar Education Program: 8-session online psychoeducation + email coaching | 8 weeks, not co-designed | Safety NR; Costs NR | Online info control | NR | Peer-based, online | Email-based |
| Sawyer 2019 | eMums Plus: nurse-led online peer group via app | 4 months, co-designed | Safety NR; Costs NR | Standard care | Review-based hypothesis | Peer-professional supported, app-based | Mobile app |
| Schlosser 2018 | PRIME: goal-setting, motivation coaches, peer community | 12 weeks, not co-designed | Safety NR; Costs NR | Waitlist | CBT, behavioral activation, mindfulness, psychoeducation | Peer-professional supported, online messaging | Online platform |
| Schulz 2016 | Self-help for SAD: clinician-guided text-based sessions | 12 weeks, not co-designed | Some participants deteriorated; Costs NR | Waitlist & group treatment | CBT | Peer-professional supported, online | Text-based sessions |
| Shalaby 2022 | PSW: peer support worker face-to-face/virtual ± text messaging | 6 months, not co-designed | Safety NR; Costs NR | TxM only, PSW+TxM, TAU | CBT | Peer-based, blended | Face-to-face/virtual + messaging |
| Shorey 2019 | PIP: correspondence with trained peer volunteer via phone/email/WhatsApp | 4 weeks, not co-designed | Safety NR; Costs NR | Standard postnatal care | Review-based hypothesis | Peer-based, blended | Phone/email/messaging apps |
| Simon 2011 | MyRecoveryPlan: online recovery plan + optional peer coaching | Up to 1 year, not co-designed | Safety NR; Costs NR | Program only (no peer coaching) | Recovery-oriented | Peer-based, online | Online platform |
| Simmons 2017 | CommonGround + SDM: peer-supported decision-making tool | 26 weeks, co-designed with end users | Safety NR; Costs NR | Historical comparison | NR | Peer-based, face-to-face | Blended (face-to-face and ablet) |
| Tomasino 2017 | MoodTech: 16 lessons with CBT-based skills practice | 8 weeks, not co-designed | Safety NR; Costs NR | 8-week waitlist | CBT | Peer-professional supported, online | Web-based CBT |
| Ugarte 2022 | HOPE: Facebook group peer education | 6 weeks, not co-designed | Safety NR; Costs NR | Facebook group without peer leaders | NR | Peer-based, online | Facebook group |
| Westerhof 2019 | The stories we live by: online life review therapy with/without peer support | 12 months, not co-designed | Safety NR; Costs NR | Waitlist | Life review therapy | Peer-professional supported, online | Web-based therapy |
| Wolf 2011/Kordy 2016 | Internet-delivered augmentations of TAU | 12 months, not co-designed | 169 AEs serious; equally distributed; Costs NR | TAU | CBT | Peer-professional supported, online | Online TAU augmentation |
| Yamaguchi 2017 | SHARE: peer support prior to psychiatric consultation | 20 months, not co-designed | Safety NR; Costs NR | TAU | CommonGround approach | Peer-professional supported, blended | Blended |

**eTable 3: Risk of bias assessment results**

| Study | Sequence generation | Allocation concealment | Intention-to-treat (yes/no) | Attrition (<5%, 5-20%,  or >20%) | Selection reporting bias | Other sources of bias | Overall |
| --- | --- | --- | --- | --- | --- | --- | --- |
| Alvarez-Jimenez 2021 | Low risk | Low risk | Low risk | Low risk | Low risk | Low risk | Low |
| Bautista 2022 | Low risk | Unclear | High risk | Low risk | Low risk | Low risk | High |
| Ellis 2011 | Low risk | Unclear | High risk | Unclear | Low risk | Unclear | High |
| Finnerty 2018 | High risk | Unclear | Unclear | High risk | Low risk | Moderate risk | High |
| Gumley 2022 | Low risk | Low risk | Low risk | Low risk | Low risk | Low risk | Low |
| Hamblen 2019 | Unclear | Unclear | High risk | Unclear | Low risk | Moderate risk | High |
| Hensel 2019 | Low risk | Low risk | Low risk | Moderate risk | Low risk | Low risk | Moderate |
| Kaplan 2011 | Low risk | Unclear | Low risk | Low risk | Low risk | Low risk | Moderate |
| Kaplan 2014/ O'Shea 2019 | Low risk | Unclear | Low risk | Moderate risk | Moderate risk | Low risk | Moderate |
| Kaveladze 2024 | Low risk | Unclear | Low risk | Low risk | Low risk | Low risk | Moderate |
| Kelly 2018 | Low risk | Low risk | High risk | Low risk | Moderate risk | Moderate risk | High |
| Merza 2023 | Low risk | Low risk | Low risk | High risk | Low risk | Low risk | High |
| Morriss 2021 | Low risk | Unclear | Low risk | Moderate risk | Low risk | Low risk | Moderate |
| Ong 2024 | Low risk | Low risk | Low risk | Moderate risk | Low risk | Low risk | Moderate |
| Pfeiffer 2020 | Low risk | Unclear | Low risk | Moderate risk | Low risk | Low risk | Moderate |
| Possemato 2019 | Low risk | Unclear | Low risk | Moderate risk | Low risk | Low risk | Moderate |
| Proudfoot 2012 | Low risk | Low risk | Unclear | Low risk | Low risk | Low risk | Moderate |
| Sawyer 2019 | Low risk | Low risk | Low risk | Moderate risk | Low risk | Low risk | Moderate |
| Schlosser 2018 | Low risk | Unclear | Low risk | Low risk | Low risk | Low risk | Moderate |
| Schulz 2016 | Unclear | Unclear | Low risk | Low risk | Low risk | Low risk | Moderate |
| Shalaby 2022 | Low risk | Unclear | High risk | High risk | Low risk | Moderate risk | High |
| Shorey 2019 | Low risk | Low risk | Low risk | Unclear | Low risk | Low risk | Moderate |
| Simon 2011 | Low risk | Unclear | High risk | High risk | Low risk | Low risk | High |
| Simmons 2017 | High risk | High risk | Unclear | Unclear | Low risk | Low risk | High |
| Tomasino 2017 | High risk | High risk | High risk | Low risk | Low risk | High risk | High |
| Ugarte 2022 | Low risk | Unclear | High risk | Low risk | Low risk | Low risk | High |
| Westerhof 2019 | Low risk | Unclear | Unclear | Moderate risk | Low risk | High risk | High |
| Wolf 2011/Kordy 2016 | Low risk | Low risk | Low risk | Low risk | Low risk | Low risk | Low |
| Yamaguchi 2017 | Unclear | Unclear | High risk | Low risk | Unclear | Moderate risk | High |

**eFigure 1: Funnel Plot Assessing Small-Study Effects for Peer Support Interventions on Depressive Symptoms**


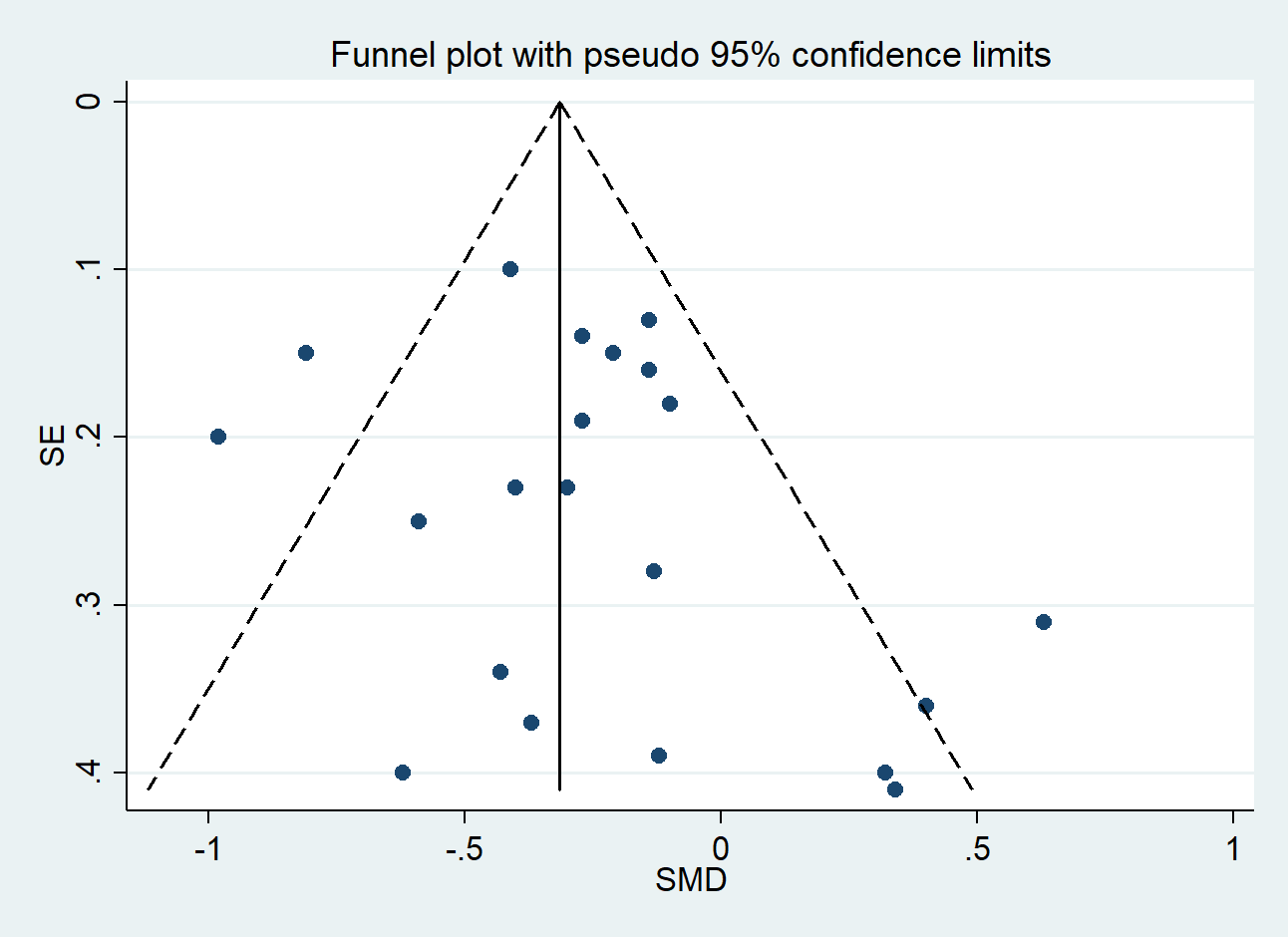


**eFigure 2: Forest Plot of Sensitivity Analyses: Effect of Peer Support Interventions on Clinical Symptoms (Excluding High Risk of Bias Studies)**


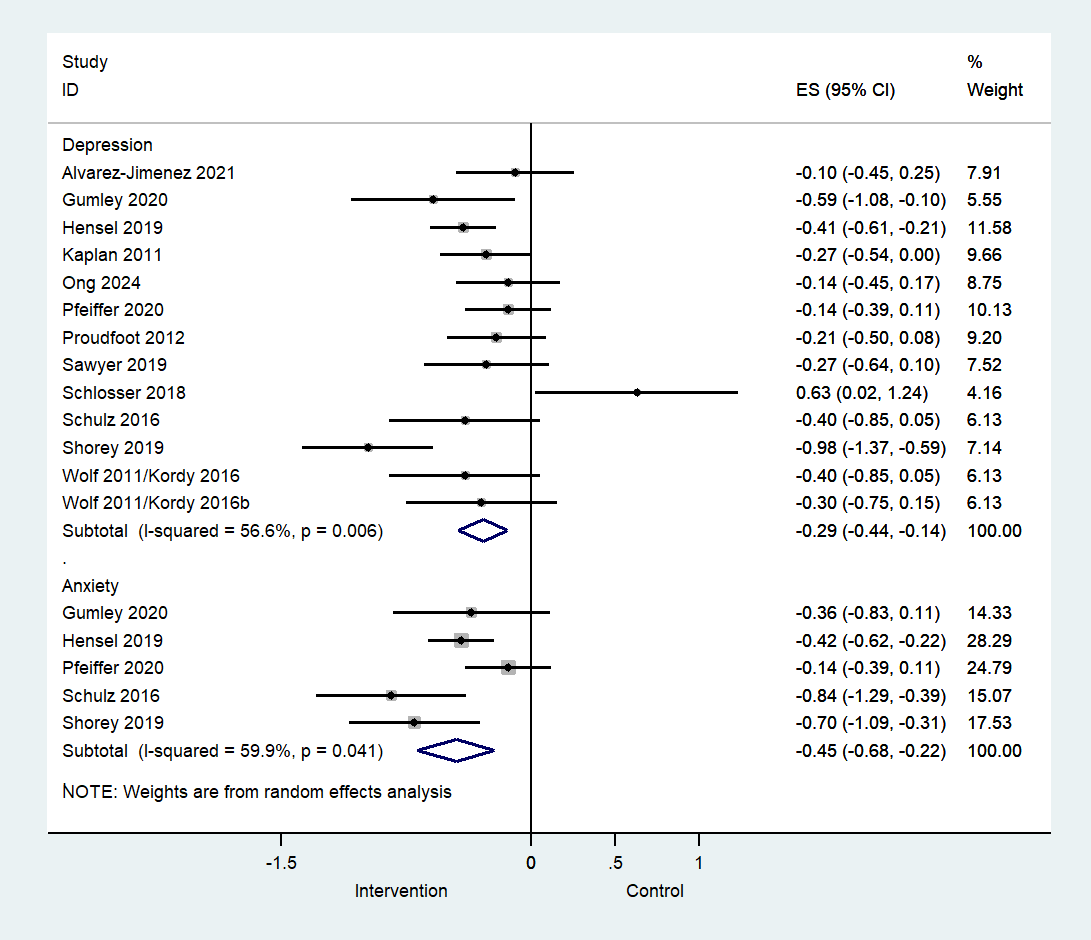


**eFigure 3: Forest Plot of Sensitivity Analyses: Effect of Peer Support Interventions on Depression ( long term follow-ups)**


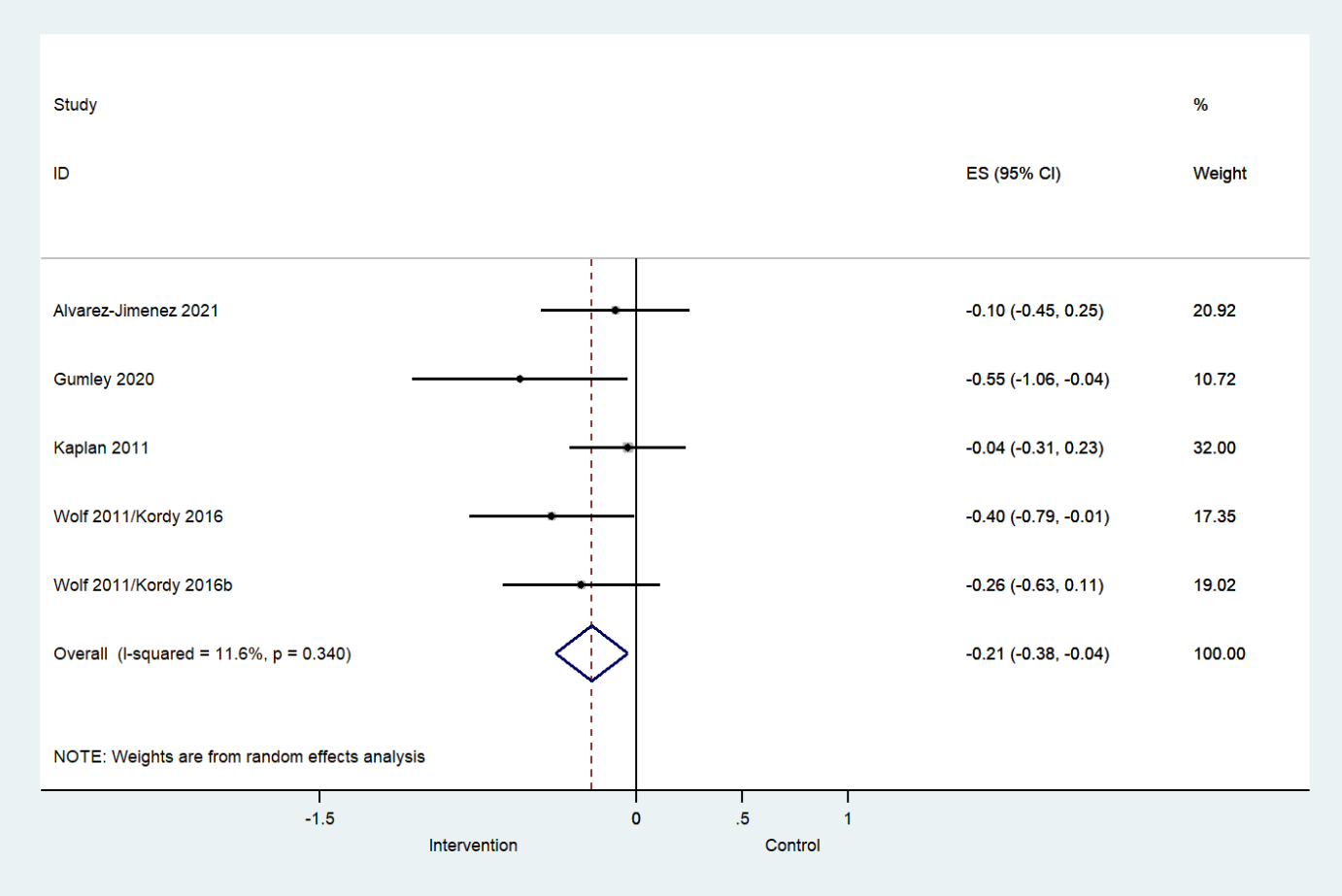


**eFigure 4: Forest Plot of Subgroup Analysis: Effect of Peer-led and Peer-Professional led Support Interventions on Depression symptoms.**


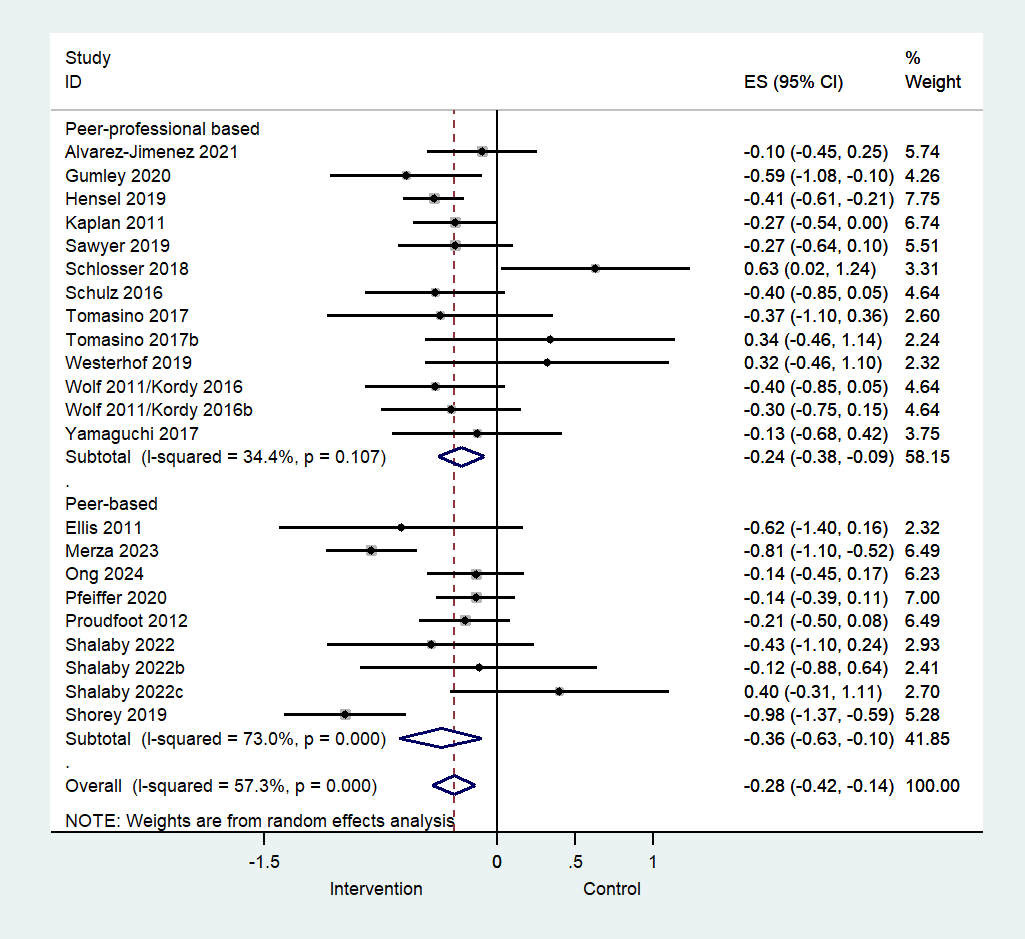


**eFigure 5: Funnel Plot Assessing Small-Study Effects for Peer Support Interventions on Anxiety Symptoms**


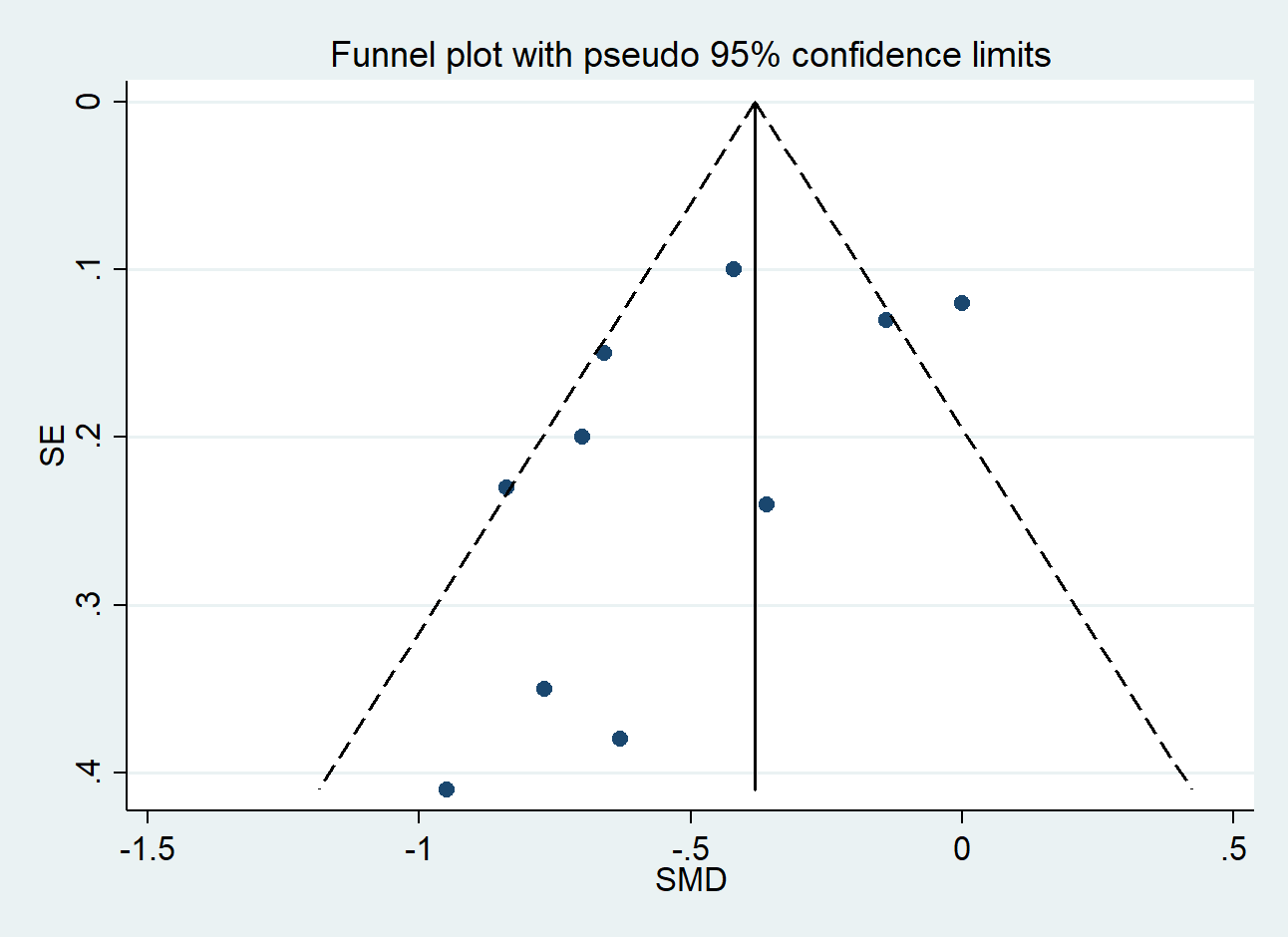


**eFigure 6: Forest Plot of Subgroup Analysis: Effect of Peer-led and Peer-Professional led Support Interventions on Anxiety symptoms.**


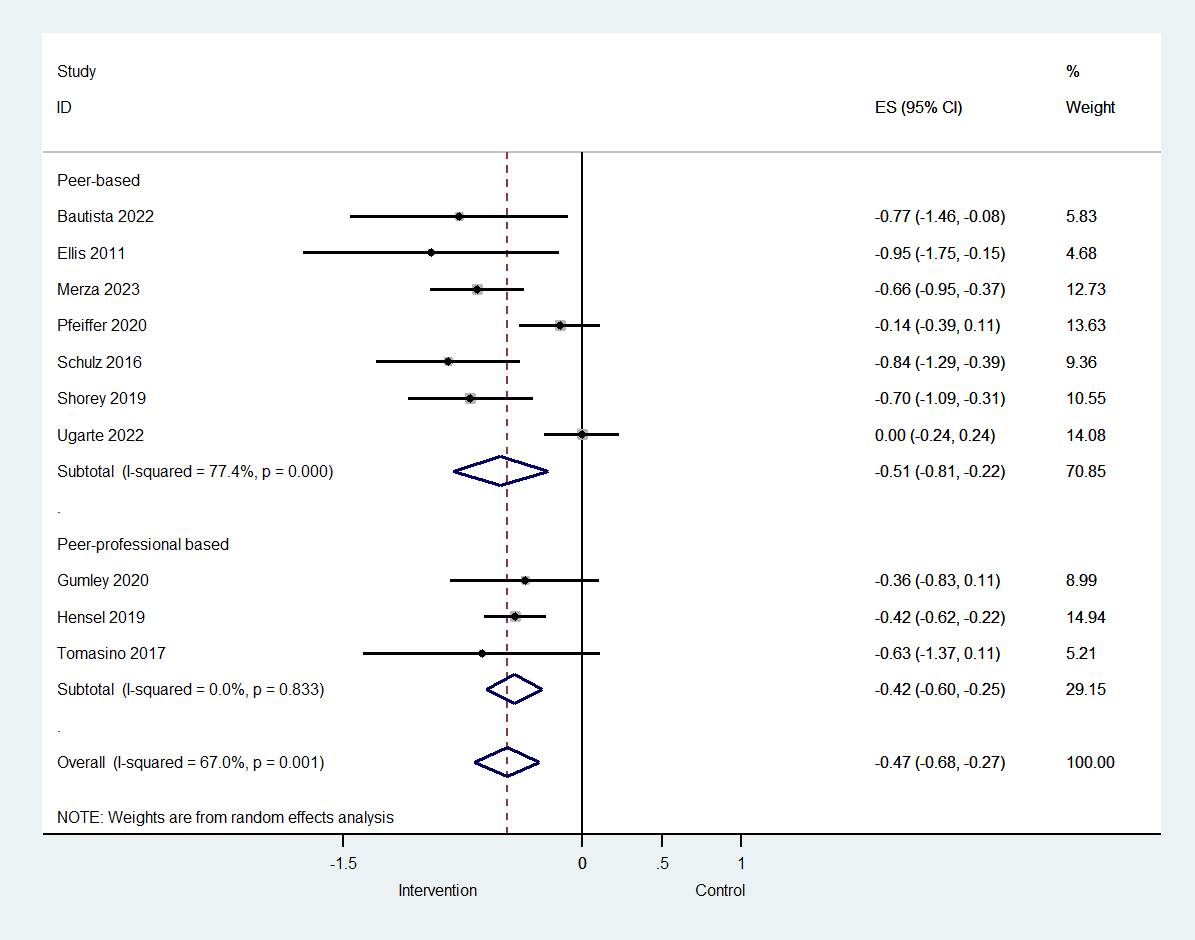


**eFigure 7: Funnel Plot Assessing Small-Study Effects for Peer Support Interventions on Social Functioning**


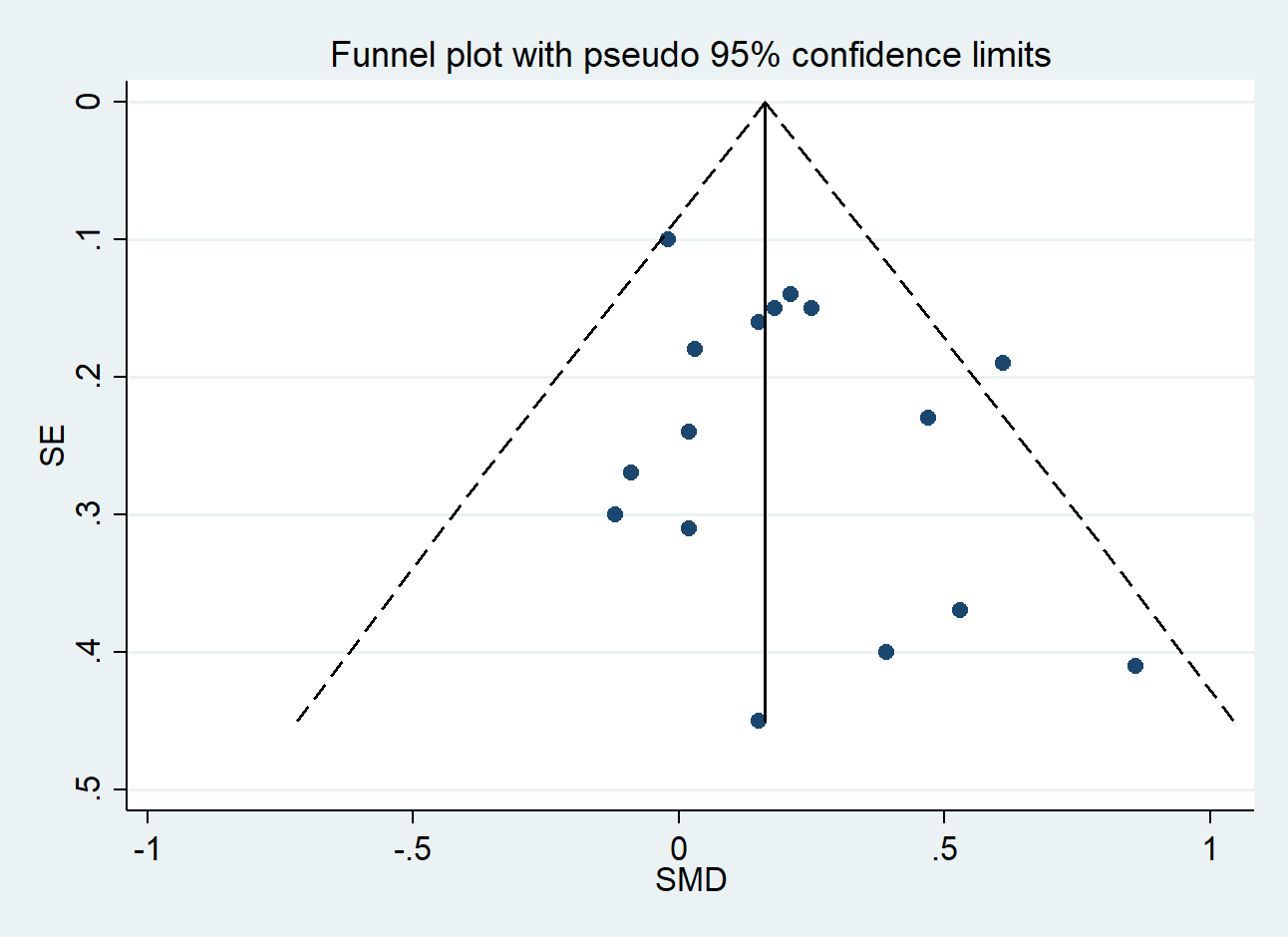


**eFigure 8: Forest Plot of Sensitivity Analyses: Effect of Peer Support Interventions on Social Functioning (Excluding High Risk of Bias Studies)**


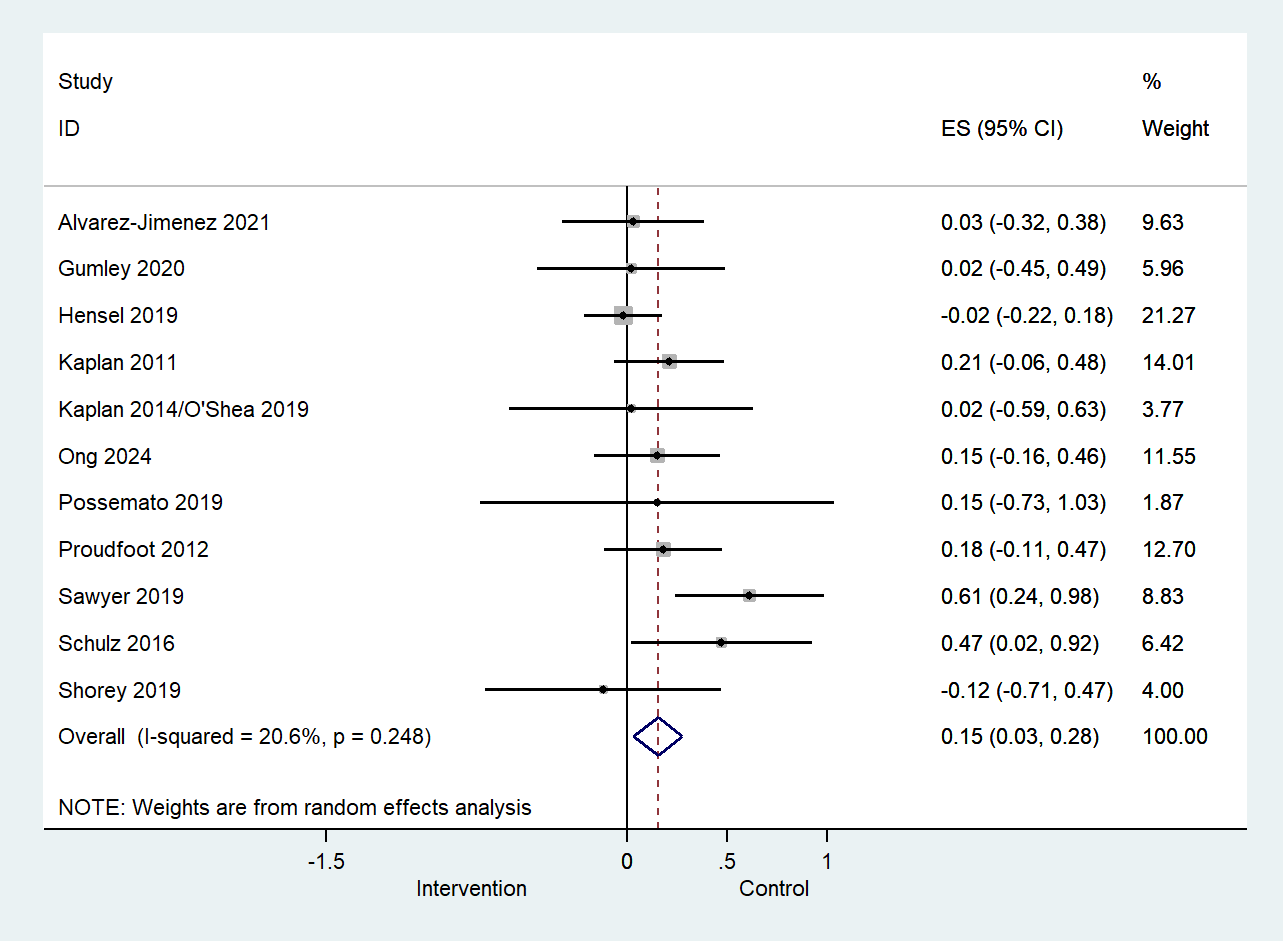


**eFigure 9: Forest Plot of Sensitivity Analyses: Effect of Peer Support Interventions on Social Functioning ( long term follow-ups)**

**
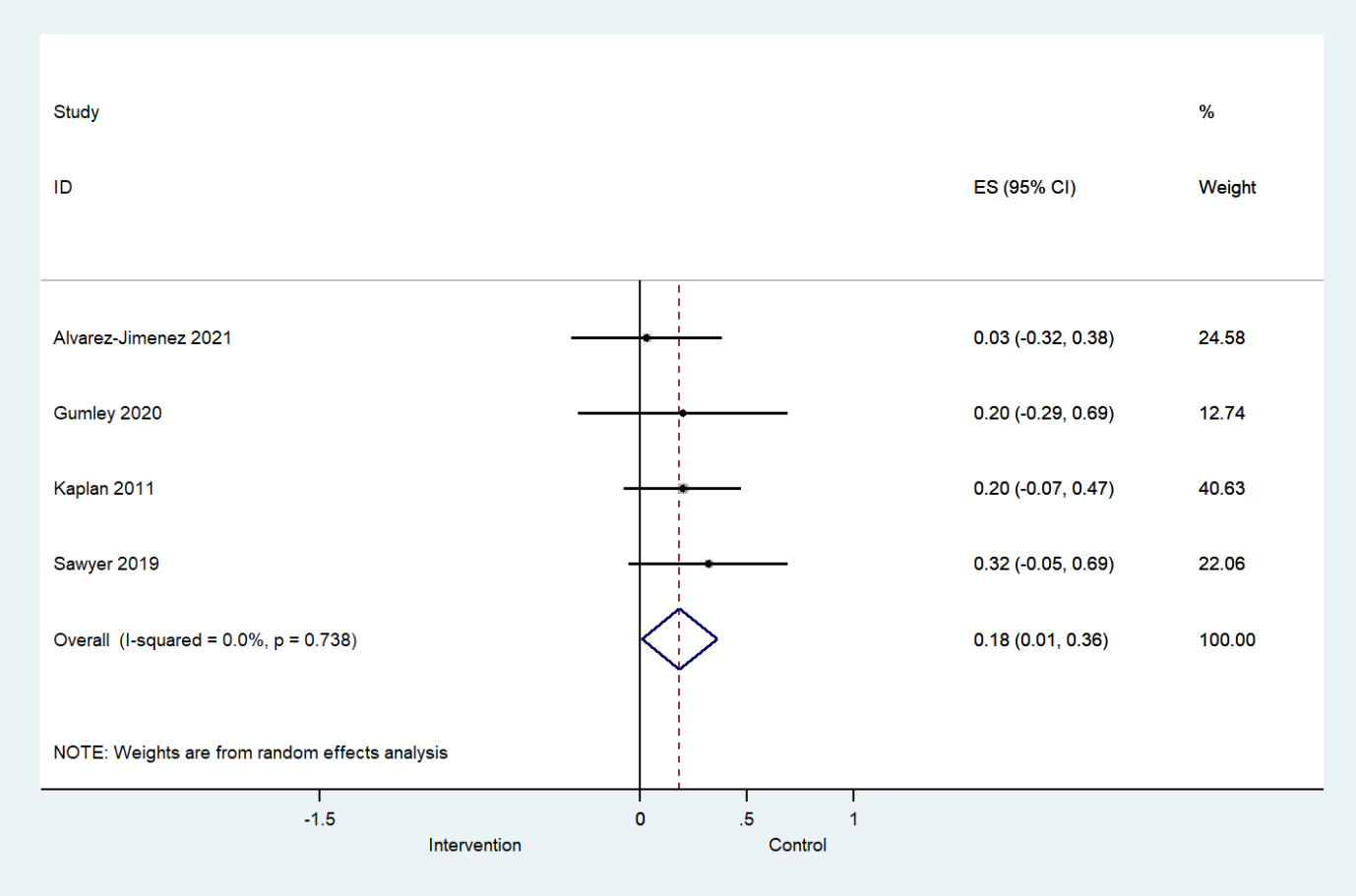
**

**eFigure 10: Forest Plot of Subgroup Analysis: Effect of Peer-led and Peer-Professional led Support Interventions on Social Functioning.**


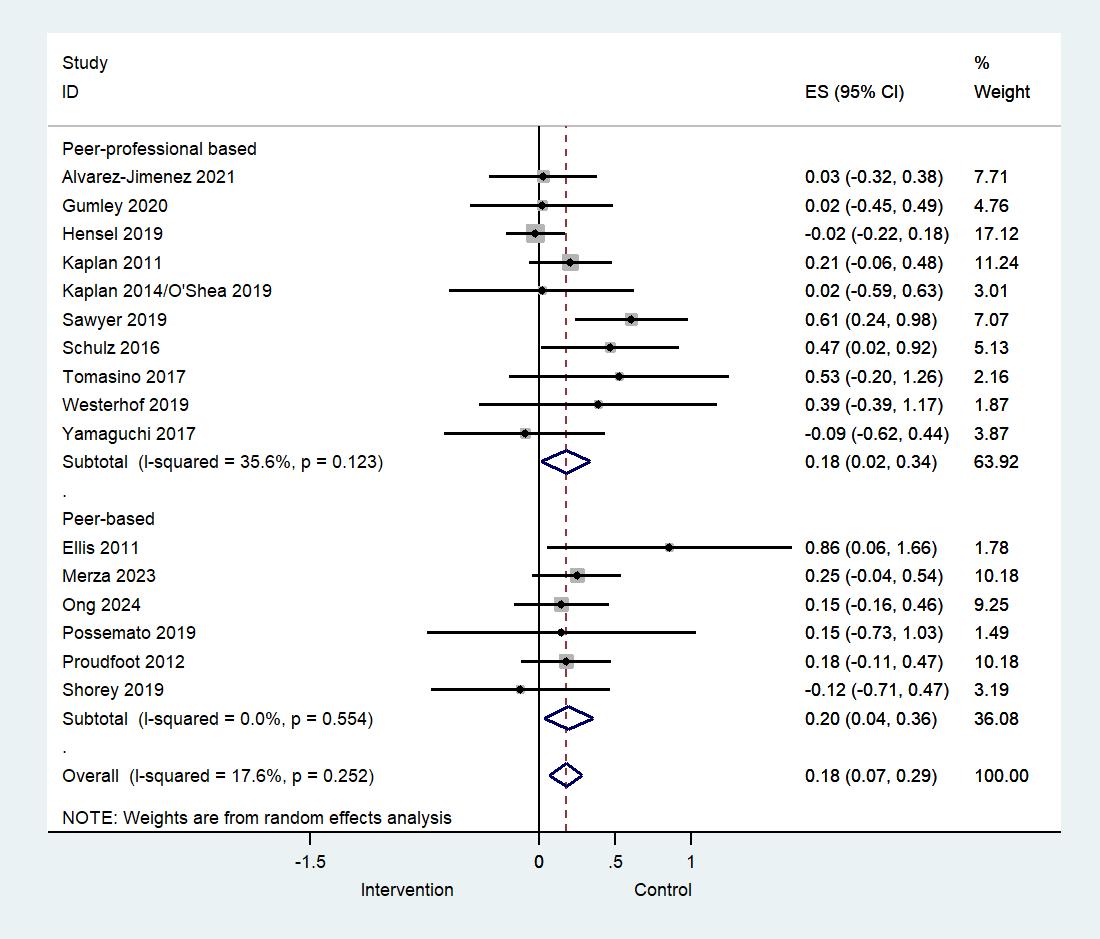


**eFigure 11: Forest Plot of the Effect of Peer Support Interventions on Treatment Engagement Aspects**


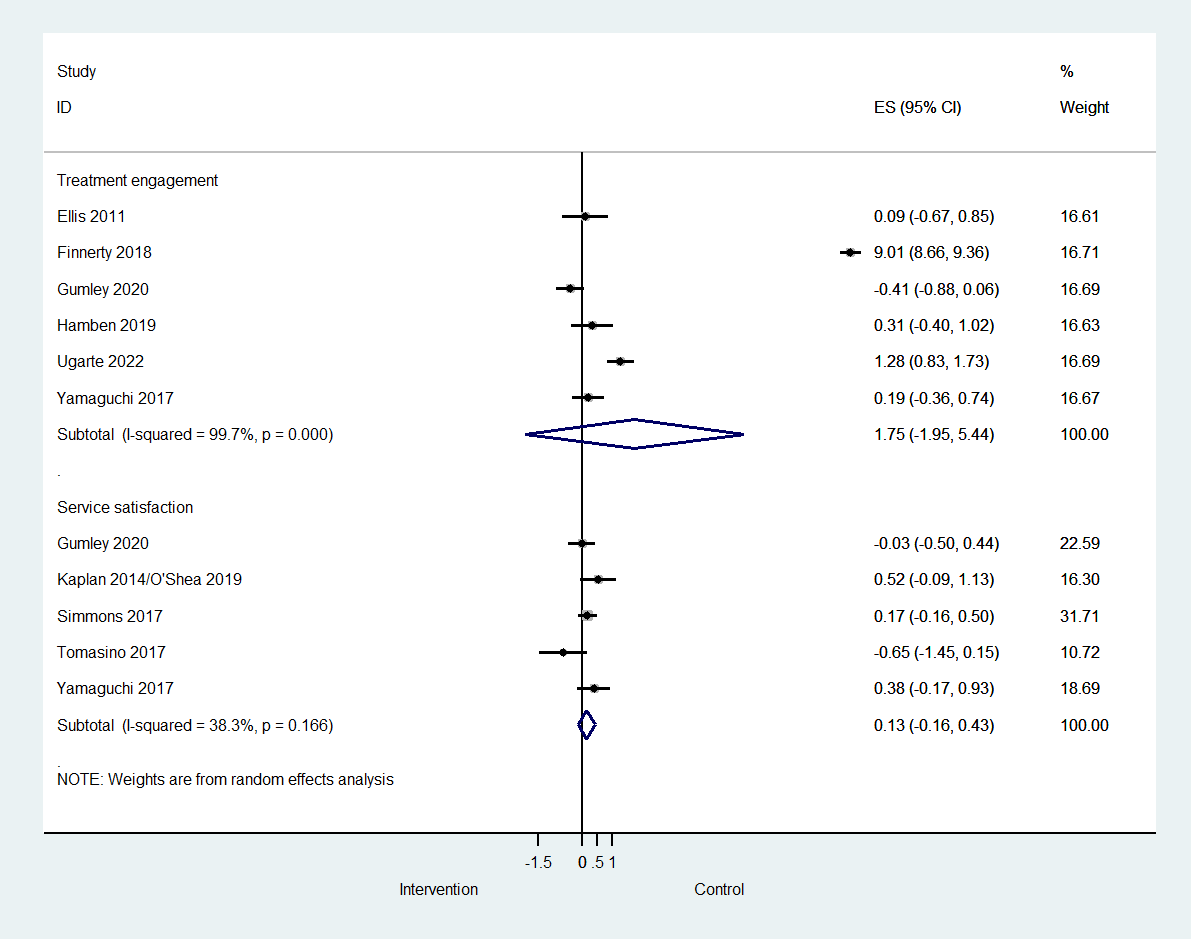

Supplement: online supplemental file 1 [file bmjment-29-1-s001.docx]
